# Evolving in the highlands: the case of the Neotropical Lerma live-bearing *Poeciliopsis infans* (Woolman, 1894) (Cyprinodontiformes: Poeciliidae) in Central Mexico

**DOI:** 10.1186/s12862-018-1172-7

**Published:** 2018-04-20

**Authors:** Rosa Gabriela Beltrán-López, Omar Domínguez-Domínguez, Rodolfo Pérez-Rodríguez, Kyle Piller, Ignacio Doadrio

**Affiliations:** 1 Programa Institucional de Doctorado en Ciencias Biológicas, Universidad Michoacana de San Nicolás de Hidalgo, Morelia, Michoacán Mexico; 2 Laboratorio de Ictiología, Centro de Investigaciones Biológicas, Universidad Autónoma del Estado de Morelos, Cuernavaca, Morelos Mexico; 3 Laboratorio de Biología Acuática, Facultad de Biología, Universidad Michoacana de San Nicolás de Hidalgo, Morelia, Michoacán Mexico; 4 Laboratorio Nacional de Análisis y Síntesis Ecológica para la Conservación de Recursos Genéticos de México, Escuela Nacional de Estudios Superiores, Unidad Morelia, Universidad Nacional Autónoma de México, Morelia, Michoacán Mexico; 5 Department of Biological Sciences, Southeastern Louisiana University, Hammond, LA 70402 USA; 6 Departamento de Biodiversidad y Biología Evolutiva, Museo Nacional de Ciencias Naturales. CSIC, Madrid, Spain

**Keywords:** Endemic fishes, Tecto-volcanism, Quaternary climate, Pliocene, Biogeographical history

## Abstract

**Background:**

Volcanic and tectonic activities in conjunction with Quaternary climate are the main events that shaped the geographical distribution of genetic variation of many lineages*. Poeciliopsis infans* is the only poeciliid species that was able to colonize the temperate highlands of central Mexico. We inferred the phylogenetic relationships, biogeographic history, and historical demography in the widespread Neotropical species *P. infans* and correlated this with geological events and the Quaternary glacial-interglacial climate in the highlands of central Mexico, using the mitochondrial genes Cytochrome b and Cytochrome oxidase I and two nuclear loci, Rhodopsin and ribosomal protein S7.

**Results:**

Populations of *P. infans* were recovered in two well-differentiated clades. The maximum genetic distances between the two clades were 3.3% for *cytb*, and 1.9% for *coxI*. The divergence of the two clades occurred ca. 2.83 Myr. Ancestral area reconstruction revealed a complex biogeographical history for *P. infans*. The Bayesian Skyline Plot showed a demographic decline, although more visible for clade A, and more recently showed a population expansion in the last 0.025 Myr. Finally, the habitat suitability modelling showed that during the LIG, clade B had more areas with high probabilities of presence in comparison to clade A, whereas for the LGM, clade A showed more areas with high probabilities of presence in comparisons to clade B.

**Conclusions:**

*Poeciliopsis infans* has had a complex evolutionary and biogeographic history, which, as in other co-distributed freshwater fishes, seems to be linked to the volcanic and tectonic activities during the Pliocene or early Pleistocene. Populations of *P. infans* distributed in lowlands showed a higher level of genetic diversity than populations distributed in highlands, which could be linked to more stable and higher temperatures in lowland areas. The fluctuations in population size through time are in agreement with the continuous fluctuations of the climate of central Mexico.

**Electronic supplementary material:**

The online version of this article (10.1186/s12862-018-1172-7) contains supplementary material, which is available to authorized users.

## Background

Volcanic and tectonic activities since the Miocene have had a substantial influence on the diversification of many New World taxa [1, 2]. Paleoclimatic events since the Pliocene, mainly from the Quaternary to the present; also have influenced the distribution of many organisms by changing the climates in boreal, temperate and tropical zones. Geologic and quaternary climatic events together are the main factors that have shaped the geographical distribution of genetic variation at the species, population, and community levels in several taxa, including fishes [3–11].

The beta diversity of freshwater fishes worldwide, including 80% of all freshwater species described, demonstrates that geographical isolation of drainage basins, combined with Quaternary climate changes, provides a parsimonious explanation for present-day patterns of spatial turnover in the global freshwater fish fauna [12]. Under this context, the geographical location, complex topography, geological dynamism including extensive volcanism since the Miocene and the climatic history of central Mexico, changed during the Quaternary [8, 13], have shaped a biogeographically complex area, characterized by ecological components that have allowed for the coexistence of taxa of Neotropical and Nearctic origins, as well as endemic groups [14, 15].

The biogeographic limits of this area have been largely discussed, and even differ depending on the taxa analyzed [14, 16–18]. In general terms, central Mexico is a high plateau bounded by the Sierra Madre Oriental to the east and by the Sierra Madre Occidental to the west and crossed by the Trans Mexican Volcanic Belt (TMVB) from west to east, with elevations up to 1400–1800 MASL [18], a region also called the Mesa Central by some authors [19]. The region is characterized by a temperate climate, thus allowing for the establishment of fish species, mainly of Neartic origin. The climatic changes during the quaternary have influenced the geographical distribution of genetic lineages of terrestrial organisms in space and time, as is the case of snakes [7], lizards [8, 20, 21], birds [9], small mammals [22–24], and plants [2].

The dynamic geological processes that have occurred since the Miocene have promoted the genesis and destruction of aquatic ecosystems [25] and have been considered as the primary forces that have influenced the biogeography and the complex evolution of several taxa of freshwater organisms [26–32]. However, most of the research has focused on understanding the complex evolutionary history of freshwater fishes of Nearctic origin such as Goodeids [28, 29, 33, 34], Cyprinids [30], Catostomids [35], and a combination of taxa [36]. Only one group of species in the TMVB that are of Neotropical origin, genus *Poeciliopsis*, has been previously investigated in this manner [26].

**Fig. 1:**
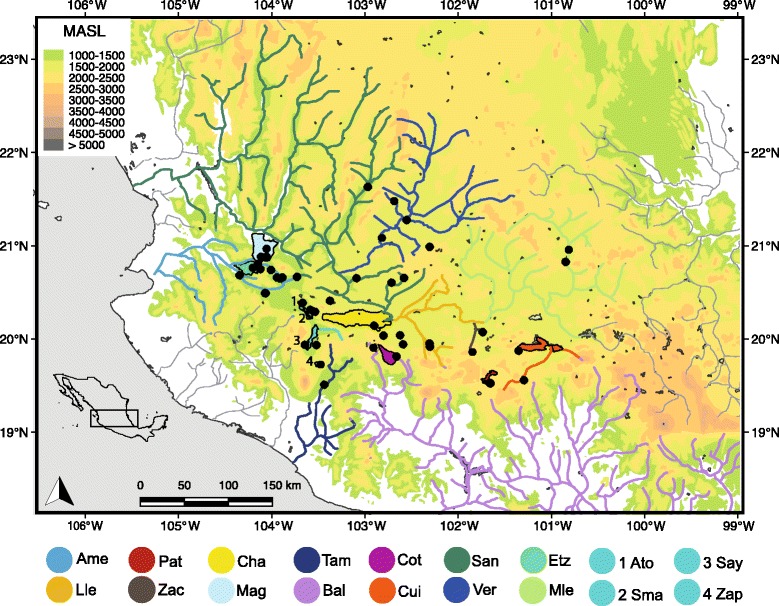
Sampling locations and the biogeographical regions where *Poeciliopsis infans* is distributed. The colors of the biogeographical regions corresponded with the colors used in the phylogenetic analyses. The codes of the biogeographical regions are as follows: (Mag) Magdalena Lake; (Etz) Etzatlan-San Marcos region; (Ver) Verde River; (Ame) Ameca River; (Ato) Atotonilco Lake as the number 1; (Sma) San Marcos Lake as the number 2; (Say) Sayula Lake as the number 3; (Zap) Zapotlan Lake as the number 4, these four lakes belong to Sayula region; (San) Grande de Santiago River; (Tam) Tamazula River; (Pat) Patzcuaro Lake; (Cha) Chapala Lake; (Bal) Balsas River; (Cot) Cotija Lake; (Lle) Lower Lerma Basin; (Mle) Middle Lerma Basin; (Zac) Zacapu region; and, (Cui) Cuitzeo Lake. The meters above see level (MASL) are indicated with level curves

The small live-bearing topminnow *Poeciliopsis infans* (Woolman, 1894) is a member of the family Poeciliidae, which has more than 220 species of tropical preferences [37]. *Poeciliopsis infans* is the only Neotropical fish species that has colonized the temperate highlands of the TMVB, including the Lerma-Santiago Basin, headwaters of the Ameca, Armeria, Coahuayana, Balsas and Panuco Basins, as well as endorheic lakes in the region (Fig. 1) [38]. *Poeciliopsis infans* is co-distributed with fishes of Nearctic origin of the families Goodeidae, Catostomidae, Ictaluridae and Cyprinidae [38].

Accordingly, since the members of the Poeciliidae are a group of Neotropical origin, most of them are adapted to tropical habitats. Furthermore, since *P. infans* is the only poeciliid species living in the temperate highlands of central Mexico, an area dominated by fish species of Neartic origin, we expect that the evolutionary history of *P. infans* could be explained by recent volcanic and tectonic activities. However, since *P. infans* is a species evolving in the marginal and temperate areas adjacent to the distribution of all of the other species *Poeciliopsis,* it may not follow the same patterns as other co-distributed species in the region.

We extensively sampled throughout the distribution of *P. infans (*Teleostomi: Poeciliidae*)*, and gathered mtDNA and nDNA sequences to infer phylogeographic variation, historical biogeography, and historical demography of the widespread *P. infans.* These data will allow us to examine the influence of geological history and Quaternary glacial-interglacial climatic events, in space and time, in the evolutionary history of Neotropical species evolving in a predominant Nearctic area.

## Methods

### Sample collection

Two hundred fifty-six specimens of *P. infans* from throughout the range were collected (Fig. 1 and Table 1) using electrofishing equipment and trawl nets. Tissue samples (fin clips) were preserved in 95% ethanol for DNA extraction, and a maximum of five specimens per site were preserved in 5% formalin. Despite intensive collection efforts, samples were not obtained from some biogeographic regions. Fish and tissue samples were deposited in the fish collection of the Universidad Michoacana de San Nicolas de Hidalgo, Mexico (SEMARNAT registration number MICH-PEC-227-07-09; for voucher numbers see Additional file 1).

**Table 1:** Samples localities and sequence information

Site	Locality	Basin	Biogeographic region	Sequences number *Cytb*/*coxI*/*S7*/*RHO*	GPS Coordinates
1	Los Venados	Magdalena	Magdalena	5/5/3/4	20° 54′ 5.5′´ N, 104° 4′ 44.8′´ W
2	Laguna	Magdalena	Magdalena	11/8/10/6	20° 54′ 14.2′´ N, 104° 1′ 11.6′´ W
3	Presa San Ignacio	Ameca	Ameca	7/6/3/6	20° 30′ 40.6′´ N, 104° 2′ 12.4′´ W
4	Chapulimita	Ameca	Ameca	2/2/2/2	20° 40′ 48.9′´ N, 103° 54′ 29.3′´ W
5	Salida presa Tecuan	Ameca	Ameca	7/6/5/5	20° 20′ 5.2′´ N, 103° 45′ 20.2′´ W
6	Manantial Los Veneros	Ameca	Ameca	2/2/2/2	20° 40′ 9.7′´ N, 103° 52′ 25.3′´ W
7	Tala, Río Salado	Ameca	Ameca	3/3/2/3	20° 41′ 12.1′´ N, 103° 41′ 36.3′´ W
8	Amatlán de cañas	Ameca	Ameca	6/5/6/5	20° 42′ 13.7′´ N, 104° 18′ 34.4′´ W
9	Teuchitlán	Cocula-La Vega	Ameca	10/6/6/4	20° 40′ 46.8′´ N, 103° 50′ 59.2′´ W
10	San Juanito de Escobedo	Laguna Colorada	Ameca	5/3/5/1	20° 45′ 37.3′´ N, 103° 59′ 39.6′´ W
11	S. M. San Julian	Verde	Verde	3/3/2/2	21° 0′ 31.7′´ N, 102° 17′ 47.9′´ W
12	San Nicolás	Verde	Verde	11/13/5/9	21° 17′ 45.4′´ N, 102° 32′ 59.7′´ W
13	Arroyo La Estancia	Verde	Verde	2/2/1/2	21° 24′ 36.3′´ N, 102° 44′ 15′´ W
14	Río Colorado	Verde	Verde	4/4/0/4	21° 5′ 6.1′´ N, 102° 52′ 10.8′´ W
15	Río Xoconostle-San Juan	Laja	Middle Lerma	3/3/3/3	20° 56′ 31.5′´ N, 100° 58′ 38′´ W
16	Manantial Andrés-Figueroa	San Marcos	Sayula	8/9/7/4	21° 17′ 45.4′´ N, 102° 32′ 59.7′´ W
17	Manantial San Marcos	San Marcos	Sayula	5/5/5/5	20° 20′ 0.4′´ N, 103° 34′ 57.6′´ W
18	Canal Presa Buena Vista	Atotonilco-Sayula	Sayula	6/7/6/3	20° 24′ 28′´ N, 103° 39′ 59.7′´ W
19	Villa corona	Atotonilco-Sayula	Sayula	3/3/3/3	20° 24′ 28.4′´ N, 103° 39′ 59.5′´ W
20	Manantial Cuyacapán	Sayula	Sayula	15/17/9/11	19° 57′ 16.6′´ N, 103° 30′ 51.7′´ W
21	Laguna de Zapotlán	Zapotlan	Sayula	2/2/1/0	19° 44′ 44.9′´ N, 103° 28′ 22.4′´ W
22	Río Las Puentes	Chapala	Chapala	6/7/7/4	20° 3′ 22.4′´ N, 102° 46′ 2.1′´ W
23	Cojumatlán	Chapala	Chapala	3/2/0/1	20° 9′ 45.7′´ N, 102° 52′ 4.6′´ W
24	Los Negritos	Chapala	Chapala	5/3/5/2	20° 3′ 36.3′´ N, 102° 36′ 46.1′´ W
25	Presa Nueva	Chapala	Chapala	5/5/5/4	19° 57′ 46.2′´ N, 102° 34′ 42.4′´ W
26	Manantial La Mintzita	Cuitzeo	Cuitzeo	6/6/5/3	19° 34′ 40′´ N, 101° 16′ 28.7′´ W
27	Ojo de Agua San Cristóbal	Cuitzeo	Cuitzeo	9/10/7/6	19° 53′ 37.4′´ N, 101° 19′ 0.5′´ W
28	Embarcadero Principal	Patzcuaro	Patzcuaro	6/7/5/1	19° 32′ 42.6′´ N, 101° 37′ 2.5′´ W
29	Urandén	Patzcuaro	Patzcuaro	8/7/6/4	19° 32′ 47.4′´ N, 101° 38′ 28.2′´ W
30	Presa Melchor Ocampo	Angulo-Lerma	Zacapu	7/7/6/2	20° 5′ 36.5′´ N, 101° 43′ 57.4′´ W
31	La Zarcita	Angulo-Lerma	Zacapu	9/10/9/4	19° 49′ 19′´ N, 101° 47′ 51′´ W
32	Laguna de Zacapu	Angulo-Lerma	Zacapu	5/5/5/1	19° 49′ 20.9′´ N, 101° 47′ 15.8′´ W
33	Atenquique	Tuxpan	Tamazula	1/1/0/1	19° 31′ 46.3′´ N, 103° 25′ 56.3′´ W
34	Puente en Jacona	Río Duero	Lower Lerma	5/5/5/4	19° 58′ 14′´ N, 102° 17′ 46.2′´ W
35	Presa La Luz	Río Duero	Lower Lerma	8/8/7/5	19° 56′ 13′´ N, 102° 17′ 56.9′´ W
36	Quitupan	Tepalcatepec	Balsas	5/5/5/3	19° 55′ 34.8′´ N, 102° 52′ 54.7′´ W
37	Presa San Juanico	Cotija	Cotija	11/9/11/6	19° 49′ 57.4′´ N, 102° 38′ 25.8′´ W
38	San Sebastián	Etzatlan-San Marcos	Etzatlan-San Marcos	2/2/2/1	20° 49′ 25′´ N, 104° 7′ 10.8′´ W
39	Presa Palo Verde	Etzatlan-San Marcos	Etzatlan-San Marcos	13/12/10/12	20° 46′ 9.6′´ N, 104° 6′ 48.2′´ W
40	Cuescomatitlán	Lago de Cajititlán	Grande de Santiago	5/5/5/2	20° 25′ 48.4′´ N, 103° 21′ 37′´ W
41	Jalpa	Juchipila	Juchipila	2/2/2/1	21° 39′ 6.5′´ N, 102° 57′ 57.8′´ W
42	San Antonio	Santiago-Chapala	Grande de Santiago	2/2/2/2	20° 40′ 27.2′´ N, 102° 33′ 19.4′´ W
43	Presa de Garabato	Santiago-Chapala	Grande de Santiago	6/6/4/5	20° 37′ 28.4′´ N, 102° 41′ 15.6′´ W
44	Río Tinajeros	Santiago-Chapala	Grande de Santiago	7/8/1/1	20° 40′ 20.7′´ N, 103° 4′ 41.9′´ W

### DNA extraction, PCR amplification and sequencing

Total genomic DNA was isolated with the Qiagen BioSprint Dneasy Tissue and Blood Kit (Qiagen, Valencia, CA, USA) following the manufacturer’s protocol. Two hundred fifty-six individuals were amplified for Cytochrome b (*cytb*: 1083 bp) and 249 individuals for Cytochrome Oxidase Subunit I (*coxI*: 631 bp), for 1771 bp of mitochondrial sequence data. The first intron, a fragment of second intron, and the second exon of the gene coding for the S7 ribosomal protein (*S7*: 859 bp), and the gene coding for the Rhodopsin protein (*RHO*: 845 bp) were amplified for 1704 bp of nuclear sequence data from 201 individuals. For this subset, individuals were chosen in order to represent all biogeographic regions and all the variation shown in mitochondrial dataset. In addition, five sequences of *cytb* were obtained from GenBank with the following accession numbers: AF412134 of Lerma River, AF412135 of Ameca River, AF412136 of Chapala Lake, AF412137 of Santiago River and AF412138 of Panuco River.

Each fragment was individually amplified using the Polymerase Chain Reaction (PCR) in volumes of 12.5 μl, containing 4.25 μl ultrapure water, 0.5 μl of each 0.2 μM primer, 6.25 μl Dream Taq Green PCR Master Mix 2× (Thermo Scientific), and 1 μl (ca. 10–100 ng) of DNA template. The specific PCR protocols of each gene are provided in Additional file 2. The recovered PCR products were purified using ExoSAP-IT (USB Corp.) and submitted to Macrogen Inc. (The Netherlands) for sequencing. Nucleotide sequences were edited and aligned in Mega v.6.06 [39], and the chromatographs were examined by eye. For the nuclear gene (*RHO*), the heterozygous genotypes were phased using DNAsp v.5.10 [40] with the algorithm provided by PHASE v.2.0 [41]. Whenever sequences of *S7* showed heterozygous positions defined by indels, a manual reconstruction of the two-allele phases was performed following the procedure described by [42]. The obtained sequences were deposited in GenBank under the follow access number: for *cytb* (MG028009 to MG028278), for *coxI* (MG028279 to MG028543), for *RHO* (MG100617 to MG100824) and for *S7* (MG366191 to MG366482), (see Additional file 1).

### Phylogenetic analyses and haplotype networks

Recombination of the nuclear genes (*RHO*, *P* = 1.0; *S7*, *P* = 0.23) was tested using the PHI test in Splitstree v.4.13 [43]. DNA sequences of each of the four genes (*cytb*, *coxI*, *S7* and *RHO*) were collapsed to haplotypes using the web-based program ALTER [44].

The phylogenetic analyses were conducted with each gene separately, with concatenated datasets for mitochondrial genes, for concatenated nuclear genes, and for the four genes combined. The phylogenetic analyses with concatenated genes were performed with the available sequences of the nuclear genes (201 specimens), the sequences of mitochondrial genes were adjusted to these number, which were representative of all biogeographic regions and account the variability found in mitochondrial genes.

The performance of the phylogenies with the four concatenated genes vs. mitochondrial genes concatenated and vs. nuclear genes concatenated were assessed using Bayes Factors (BF). Bayes Factors were calculated from the estimated harmonic means of likelihood using the sump command in MrBayes. Decisions were made based on the 2ln BF criterion, with BF > or = 10 considered as strong evidence for rejecting the null hypothesis [45].

Model selection based on Akaike information criterion (AIC) and optimal partition settings were performed using PartitionFinder v.1.1.0 [46], and recovered the best partition by assigning substitution models for each gene. The parameters of each model are provided in Additional file 3.

Gene trees were constructed with Maximum Likelihood implemented in RAxMLGUI v.1.3.1 [47, 48], using the substitution model implemented for each gene. The GTR + G + I [48] substitution model was used for the concatenated genes matrix and 10,000 bootstrap replicates with the algorithm ML + rapid bootstrap. The relative stability of clades was evaluated by 1000 non-parametric bootstrap replicates [49].

Bayesian Inference was implemented in MrBayes v.3.2.1 [50], with the substitution models for each gene obtained in PartitionFinder. Analyses were run for 10 million generations, with two independent runs implementing four Markov Chain Monte Carlo (MCMC) processes and sampling every 500 generations. We evaluated the chains for convergence with the log-likelihood (-InL) values of the two independent runs using Tracer v.1.5 [51], and discarded 10% as burn-in to construct the consensus tree. *Poeciliopsis prolifica* was used as outgroup, based on the results of a previous study [26].

In order to determine the geographic distribution of haplotypes for all populations of *P. infans* for nuclear genes, we reconstructed two independent TCS haplotype networks (a phylogenetic network estimation using statistical parsimony) for *RHO* and *S7* sequences using PopArt v.1.7 (http://popart.otago.ac.nz).

### Divergence time estimation and genetic distances

The program BEAST v.1.8.1 [52] was used to estimate the most recent common ancestor for clades within *P. infans.* This analysis was carried out with a subset of 145 sequences that include all different haplotypes for all genes. Because the lack of fossil data for *Poeciliopsis*, the molecular clock was calibrated using the mutation rate of *cytb* in teleosts of 0.76–2.2%/million years [53–55]. Since the mutation rate is not available for the other genes, they were included in the analysis without calibration information.

The model parameters were unlinked across *cytb*, *coxI*, *S7* and *RHO* genes and substitution models were set according to the selected model for each gene by PartitionFinder v. 1.1.0 [46]. We applied a lognormal relaxed clock (Uncorrelated) model on branch length [56]. We selected the tree prior Coalescent: Extended Bayesian Skyline Plot [57], and estimated a starting tree using the random method. A MCMC analysis with 50 million of generations was conducted, and sampled every 1000 generations. We assessed whether parameter values had reached effective sample size and convergence in Tracer v.1.5. [51]. Finally, the maximum clade credibility tree was built, discarding the first 10% of the trees as burn-in, using Tree Annotator v.1.8.1. [52].

Uncorrected genetic distances were calculated among the recovered groups in the phylogenetic trees for each mitochondrial gene (*cytb*, *coxI*), and between all individuals for *S7* and *RHO* in Mega v.6.06 [39]. A bootstrapping process was performed with 1000 repetitions.

### Genetic diversity and population structure

For each gene (*cytb*, *coxI*, *S7* and *RHO*), the number of haplotypes (H), polymorphic sites (S), nucleotide (π) and haplotype (h) diversities were obtained to estimate genetic diversity levels in all populations of *P. infans*. To examine genetic differentiation at different hierarchical levels, as well as geographical patterns of population subdivision, an analysis of molecular variance (AMOVA) was conducted. The AMOVAs were implemented for the four separate genes and groupings according to: 1) inferred clades in phylogenetic analyses, 2) according to the biogeographic regions sensu [28] and, a third analysis was performed without a priori groupings. The analyses were conducted using 10,000 permutations to assess significance values. All genetic diversity analyses and AMOVAs were performed in Arlequin v.3.5.1 [58].

### Ancestral area reconstruction

The ancestral area reconstruction for *P. infans* was estimated using the statistical Dispersal-Vicariance (S-DIVA) method [59], and the Dispersal-Extinction-Cladogenesis (DEC) model [60, 61] as implemented in RASP v.3.2 software [62]. These methods use statistical approaches to reconstruct biogeographic history, and allowed us to compare both results. The ultrametric and dichotomous tree obtained for *cytb* in BEAST was used as the tree topology on which ancestral areas were mapped. For both analyses, the maximum number of areas was limited to two. For these analyses, the distributional area of *P. infans* was divided into 15 biogeographic regions: (Mag) Magdalena Lake; (Etz) Etzatlán-San Marcos region; (Ver) Verde River; (Ame) Ameca River; (San) Grande de Santiago River; (Tam) Tamazula River; (Pat) Patzcuaro Lake; (Cha) Chapala Lake; (Bal) Balsas River; (Cot) Cotija Lake; (Lle) Lower Lerma River; (Mle) Middle Lerma River; (Zac) Zacapu Lake and (Cui) Cuitzeo Lake, and, within Sayula region are considered the follow Lakes: (Ato) Atotonilco, (Sma) San Marcos, (Say) Sayula and (Zap) Zapotlan [28, 29].

### Historical demography

The population size fluctuations through time were tested with a Coalescent Bayesian Skyline Plot (BSP) analysis [63] as implemented in BEAST v.1.8.1 [52]. This analysis only was implemented with sequences of *cytb* due the higher number of available sequences. The substitution rate was the same as the divergence time analysis and the substitution model was set according to the select model by PartitionFinder v. 1.1.0 [46].

An uncorrelated relaxed clock model was set a priori, and 70 million generations were run, sampled every 1000 generations. Convergence was assessed with Tracer v.1.5 [51]. The first 10% of the states were discarded as burn-in.

### *Poeciliopsis infans* Distribution modelling

To evaluate the concordance between the historical demography obtained in BSP analyses and the potential distribution of *P. infans* in the past, we carried out a species distribution modelling analyses at different temporal scales [64]. The estimations of the current and past population distribution were inferred with MaxEnt v.3.3.1 [65]. Geographical coordinates of 162 sites registered in the database of the Colección de Peces de la Universidad Michoacana de San Nicolás de Hidalgo were used as presence data (see Additional file 4). For the environmental data, we used 19 bioclimatic variables downloaded from WORLDCLIM database [66], http://www.worldclim.org, at a resolution of 30 arc-seconds for Last Inter Glacial (LIG) and 2.5 min for Last Glacial Maximum (LGM). The WORLDCLIM variables represent biologically meaningful summaries of precipitation and temperature in the present (1950–2000), and for the past, the Community Climate System Model (CCSM) for the LGM: 0.025 Myr, and the LIG: 0.15–0.10 Myr periods. To construct the models, we employed a logistic output [65], and the default settings. The value of the regularization multiplier was tested for 0.5, 1.0, 1.5 and 2.0, but the highest AUC value was for a regularization multiplier of one, and this value was used in all analyses. The model was run with 100 subsample replicates estimating mean habitat suitability values (S).

To evaluate if the performance of all distribution models was better than one, a random model was assessed using 75% of the presence data to run the model and the remaining 25% for statistical testing. In addition, the area under the receiver operating characteristic curve (AUC) was estimated to assess the accuracy of the models. A jackknife test of variables of importance was conducted to evaluate the relative importance of each climate variable [67]. Variables contributing the least to the model or those highly correlated [68] were removed for each model. The correlation of variables was evaluated through the response curves, which reflect the dependencies induced by correlations between the selected variable and other variables.

## Results

### Phylogenetic relationships and haplotype networks

The ambiguously aligned positions that showed the sequences of the S7 gene are see in Additional file 5. The BF comparison indicated that the analyses using the four concatenated genes provided a better explanation of the data than the mitochondrial genes concatenated or nuclear genes concatenated (BF of four genes concatenated *vs* mitochondrial genes concatenated = 1.87 and BF of four concatenated genes vs nuclear genes concatenated = 2.2).

**Fig. 2:**
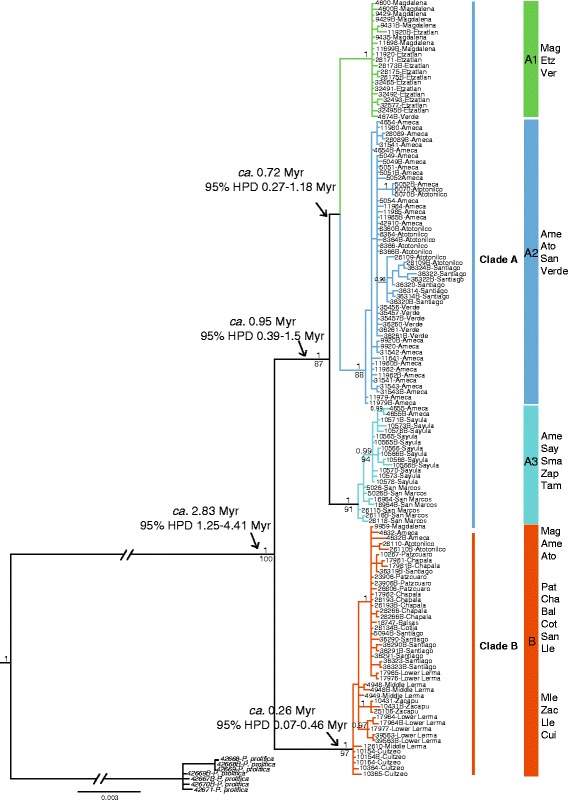
The Bayesian inference tree of *Poeciliopsis infans* from concatenated sequences of two mitochondrial (*cytb*, *coxI*) and two nuclear genes (*S7* and *RHO*). Bayesian posterior probability (> 0.9; above the branches) and maximum likelihood bootstrap values (> 80%; below the branches) are indicated. The divergence time estimations are shown with 95% HPD

The phylogenetic results for both analyses (Maximum likelihood and Bayesian Inference) recovered two well differentiated and well supported clades for each mitochondrial gene (*cytb*: 1083 bp and *coxI*: 631 bp) (Additional files 6 and 7), for the concatenated mitochondrial genes (*cytb*, *coxI*: 1771 bp) (Additional file 8), and for all concatenated genes (*cytb*, *coxI*, *S7* and *RHO:* 3475 bp; Fig. 2).

In general terms, clade A clustered populations of the biogeographic regions of lowlands of TMVB, while; clade B clustered populations of the biogeographic regions of the highlands of the TMVB. However, one sample from Ameca, Magdalena and Sayula regions respectively were grouped in clade B. (Figs. 1 and 2).

Clade A clustered individuals of seven biogeographic regions is better in three sub-clades: sub-clade A1 included individuals from the Etzatlan-San Marcos region, Magdalena Lake and few individuals of Verde River; sub-clade A2 clustered individuals of the Ameca, Verde, and Grande de Santiago Rivers and the Atotonilco Lake of the Sayula region; while the sub-clade A3 clustered individuals of the Sayula, San Marcos and Zapotlan Lakes within the Sayula region, as well as samples from the Ameca River basin and Tuxpan River of the Tamazula biogeographic region. These three sub-clades were well supported in the Bayesian inference analysis, but the bootstrap support for sub-clade A1 was low (87%) (Fig. 2).

The second clade, clade B, clustered individuals of the Middle and Lower Lerma, Grande de Santiago and Balsas Rivers, as well as Cuitzeo, Patzcuaro, Chapala, Cotija and Zacapu Lakes. Also, three samples corresponding to regions that were clustered in clade A were recovered in clade B and corresponded to Magdalena and Atotonilco Lakes, and the Ameca River (Fig. 2). The phylogeny based on each nuclear gene and with both concatenated nuclear genes failed to recover resolved relationships (Additional files 9, 10 and 11).

For the haplotype networks based on the nuclear genes, two haplogroups (A and B) were recovered and these are highly congruent with the concatenated gene phylogeny. There are, however, shared haplotypes between them, with *RHO* network sharing the most haplotypes between groups. The first haplogroup (A), grouped individuals of Ameca, Grande de Santiago, Sayula, Magdalena, Verde and Etzatlan-San Marcos regions, for *S7*, one individual of the Chapala region was found in this haplogroup. The second haplogroup (B) for both nuclear genes, grouped individuals of the Lower and Middle Lerma, Grande de Santiago, Balsas, Zacapu, Cuitzeo, Patzcuaro, Cotija and Chapala regions. Some individuals of the Sayula region were grouped in the haplogroup B for both genes (see Additional file 12).

### Divergence time estimation and genetic distances

**Fig. 3:**
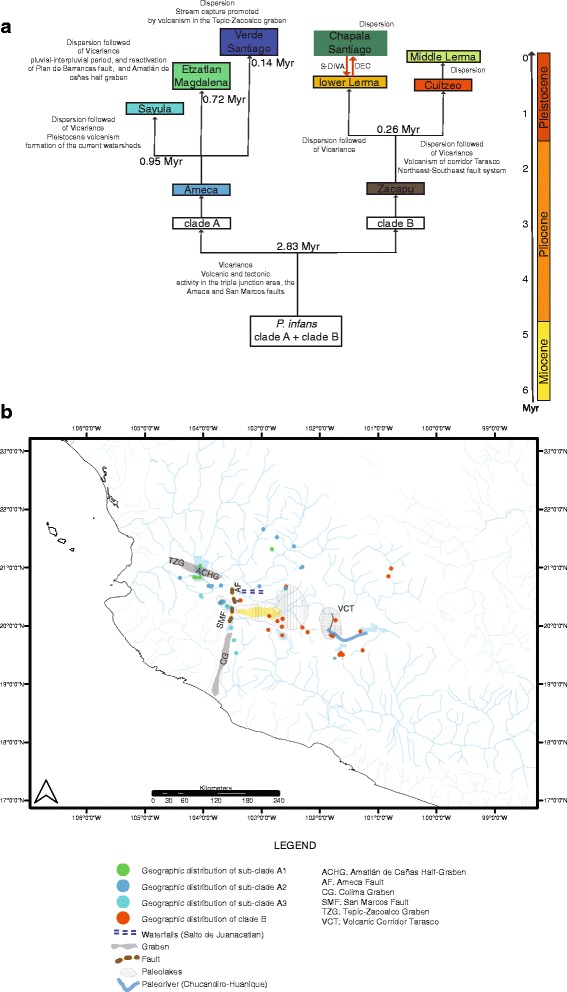
**a** Divergence times, biogeographical events in *Poeciliopsis infans*, **b** geographic distribution of inferred clades and sub-clades across of TMVB of *P. infans*, and tectonic, volcanic and hydrographical features of the TMVB

The first isolation event in *P. infans,* separating clades A and B, was estimated to have occurred near the middle Pliocene and middle Pleistocene ca. 2.83 Myr (95% HPD: 1.25–4.41 Myr). The first separation event of the three sub-clades within clade A was estimated to have occurred during the Pleistocene period ca. 0.95 Myr (95% HPD: 0.39–1.5 Myr), whereas the rest of the isolation events were calculated in less of a million of years (Figs. 2 and 3).

The uncorrected mean genetic distances (*p*-distance) calculated between clade B and sub-clades A1, A2 and A3 for the mitochondrial genes ranged from 0.8–3.3% for *cytb*; and 0.7–1.9% for *coxI* (Table 2). The minimum genetic distances found for *cytb* were between sub-clade A1 and sub-clade A2 (0.8%), and the maximum distances were between sub-clade A3 and clade B (3.3%). Based on *coxI* the minimum genetic distances were observed between sub-clade A2 and sub-clade A3 (0.7%), and the maximum were between sub-clade A1 and clade B (1.9%). Between the three sub-clades of clade A the genetic distances were 0.8–1.1% for *cytb* and 0.7–1.3% for *coxI* (Table 2). For nuclear genes the genetic distances between all individuals included both alleles for each sequence, ranged between 0.0–0.5% for *RHO* and between 0.0–0.6% for S7.

**Table 2:** Uncorrected genetic distances presented in proportion

*cytb*/*coxI*	Clade A			Clade B
	Sub-clade	Sub-clade	Sub-clade	
	A1	A2	A3	
Sub-clade A1	0.001/0.000	0.008	0.010	0.031
Sub-clade A2	0.013	0.001/0.001	0.011	0.031
Sub-clade A3	0.009	0.007	0.001/0.000	0.033
Clade B	0.019	0.016	0.016	0.002/0.002

Genetic distances within recovered clades and sub-clades of *Poeciliopsis infans* based on *cytb* (to the left of the diagonal) and *coxI* (to the right of the diagonal) and between recovered groups in phylogenetic analyses based on *cytb* (above the diagonal) and *coxI* (below the diagonal) genes

### Genetic diversity and population structure

The highest haplotype diversity was found in Cuitzeo (*h* = 0.81) for *cytb* and in the Verde River (*h* = 0.62) for *coxI*, followed by the Ameca River Basin for both genes (*h* = 0.75 for *cytb* and 0.42 for *coxI*), while, null haplotype diversity were found in the Patzcuaro, Cotija and Balsas regions. Atotonilco Lake, within the Sayula region and the Verde River exhibited the highest nucleotide diversity for *cytb* and *coxI* (*π* = 0.006, *π* = 0.004) respectively (Table 3).

**Table 3:** Genetic diversity for each biogeographic region of *Poeciliopsis infans* based on mitochondrial DNA data (*cytb* and *coxI*)

*cytb* – *coxI*					
Biogeographic region	N	S	H	*π*	*h*
Magdalena	15–12	1–0	2–1	0.000–0.000	0.133–0.000
Ameca	37–30	18–5	9–3	0.001–0.001	0.752–0.427
Middle Lerma	3–3	3–0	3–1	0.001–0.000	1.000–0.000
San Marcos (Sayula)	13–14	3–1	4–2	0.000–0.000	0.525–0.362
Atotonilco (Sayula)	9–9	34–0	2–1	0.006–0.000	0.220–0.000
Sayula (Sayula)	15–17	4–2	5–3	0.001–0.000	0.695–0.227
Cuitzeo	15–16	8–2	6–3	0.001–0.000	0.819–0.241
Patzcuaro	14–14	0–0	1–1	0.000–0.000	0.000–0.000
Zacapu	21–22	8–2	6–2	0.001–0.000	0.557–0.173
Etzatlan-San Marcos	19–16	7–0	7–1	0.000–0.000	0.608–0.000
Chapala	19–17	2–0	3–1	0.000–0.000	0.292–0.000
Balsas	5–5	0–0	1–1	0.000–0.000	0.000–0.000
Cotija	11–9	0–0	1–1	0.000–0.000	0.000–0.000
Verde	19–21	9–9	2–3	0.002–0.004	0.280–0.620
Grande de Santiago (before SJ)	12–11	3–1	3–2	0.000–0.000	0.547–0.388
Grande de Santiago (after SJ)	10–11	2–1	3–2	0.000–0.000	0.375–0.250
Lower Lerma	13–13	3–4	2–3	0.000–0.001	0.282–0.410

N, sample size, S, polymorphic sites, H, number of haplotypes, *π,* nucleotide diversity *h,* haplotype diversity. SJ = Salto de Juanacatlan

For the nuclear genes, the highest haplotype diversity was found in the Atotonilco (*h* = 0.97), and Ameca (*h* = 0.9) for *S7*, and the Middle Lerma (*h* = 0.73) for *RHO*. Absence of haplotype diversity was found for the Middle Lerma for *S7* and Cuitzeo for *RHO*. The populations of Atotonilco (*π* = 0.01) and the Middle Lerma (*π* = 0.001) exhibited the highest nucleotide diversity for *S7* and *RHO* respectively (Table 4). When the AMOVA was performed without groups a priori, the highest variation for all genes were among populations (*cytb*: 91.17%, *coxI*: 96.07%, *S7*: 69.5% and *RHO*: 60.64%) and not within populations. The AMOVA for all genes showed a high percentage of variation when populations were grouped according to the recovered clades and sub-clades from the phylogenetic analyses (*cytb*: 92.24%, *coxI*: 91.03%, *S7*: 45.48% and *RHO*: 53.21%), but not when populations were grouped according to the biogeographic regions [18] (*cytb*: 31.31%, *coxI*: 27.09%, *S7*: -99.28% and *RHO*: 53.21) (Tables 5 and 6).

**Table 4:** Genetic diversity for each biogeographic region of *Poeciliopsis infans* based on nuclear DNA data (*S7* and *RHO*)

*S7* – *RHO*					
Biogeographic region	N	S	H	*π*	*h*
Magdalena	24–18	10–6	6–5	0.002–0.000	0.713–0.405
Ameca	50–54	29–2	22–3	0.004–0.000	0.956–0.265
Middle Lerma	6–6	0–2	1–3	0.000–0.001	0.000–0.733
San Marcos (Sayula)	24–20	1–1	2–2	0.000–0.000	0.159–0.505
Atotonilco-Sayula (Sayula)	18–10	39–2	14–2	0.010–0.000	0.973–0.200
Sayula (Sayula)	20–22	7–2	6–3	0.002–0.000	0.790–0.177
Cuitzeo	24–18	2–0	3–1	0.000–0.000	0.358–0.000
Patzcuaro	22–10	1–1	2–2	0.000–0.000	0.173–0.466
Zacapu	40–14	4–2	5–2	0.000–0.000	0.315–0.142
Etzatlan-San Marcos	32–30	10–3	14–3	0.001–0.000	0.774–0.131
Chapala	34–26	8–2	8–3	0.001–0.000	0.711–0.280
Balsas	10–6	4–1	4–2	0.001–0.000	0.644–0.333
Cotija	22–18	2–1	3–2	0.000–0.000	0.450–0.529
Verde	14–32	11–1	6–2	0.004–0.000	0.822–0.000
Grande de Santiago	32–26	15–3	16–4	0.002–0.001	0.834–0.600
Lower Lerma	26–18	15–2	5–3	0.001–0.000	0.461–0.307

N, sample size (included the two alleles of each sequence), S, polymorphic sites, H, number of haplotypes, *π,* nucleotide diversity *h,* haplotype diversity

**Table 5:** Analyses of molecular variance (AMOVA) of the mitochondrial data for select groups of *Poeciliopsis infans* at different hierarchical arrangements

Testing assumptions	Source of variation	% of variance	Fixation index	*P*- value
*Cytb*				
Grouped according to recovered clades and sub-clades	Among groups	92.24	*Φ*_CT_: 0.92	< 0.0001
	Among populations within groups	3.82	*Φ*_SC_: 0.49	< 0.0001
	Within populations	3.94	*Φ*_ST_: 0.96	< 0.0001
	Total	100		
Biogeographic regions	Among groups	31.31	*Φ*_CT_: 0.31	*ns*
	Among populations within groups	63.5	*Φ*_SC_: 0.94	< 0.0001
	Within populations	5.19	*Φ*_ST_: 0.94	< 0.0001
	Total	100		
Without grouping a priori	Among populations	91.17	*Φ*_ST_: 0.91	< 0.0001
	Within populations	8.83		
	Total	100		
*CoxI*				
Grouped according to recovered clades and sub-clades	Among groups	91.03	*Φ*_CT_: 0.91	< 0.0001
	Among populations within groups	6.01	*Φ*_SC_: 0.66	< 0.0001
	Within populations	2.96	*Φ*_ST_: 0.97	< 0.0001
	Total	100		
Biogeographic regions	Among groups	27.09	*Φ*_CT_: 0.27	*ns*
	Among populations within groups	69.01	*Φ*_SC_: 0.94	< 0.0001
	Within populations	3.9	*Φ*_ST_: 0.96	< 0.0001
	Total	100		
Without grouping a priori	Among populations	96.07	*Φ*_ST_: 0.96	< 0.0001
	Within populations	3.93		
	Total	100		

*ns* = not significant

**Table 6:** Analyses of molecular variance (AMOVA) of the nuclear data for select groups of *Poeciliopsis infans* at different hierarchical arrangements

Testing assumptions	Source of variation	% of variance	Fixation index	*P*- value
*S7*				
Grouped according to recovered clades and sub-clades	Among groups	45.48	*Φ*_CT_: 0.45	< 0.0001
	Among populations within groups	28.26	*Φ*_SC_: 0.51	< 0.0001
	Within populations	26.26	*Φ*_ST_: 0.73	< 0.0001
	Total	100		
Biogeographic regions	Among groups	-99.28	*Φ*_CT_:-0.99	*ns*
	Among populations within groups	166.42	*Φ*_SC_:0.83	< 0.0001
	Within populations	32.86	*Φ*_ST_:0.67	< 0.0001
	Total	100		
Without grouping a priori	Among populations	69.5	*Φ*_ST_: 0.69	< 0.0001
	Within populations	30.5		
	Total	100		
*RHO*				
Grouped according to recovered clades and sub-clades	Among groups	53.21	*Φ*_CT_: 0.53	< 0.0001
	Among populations within groups	12.42	*Φ*_SC_: 0.26	< 0.0001
	Within populations	34.37	*Φ*_ST_: 0.65	< 0.0001
	Total	100		
Biogeographic regions	Among groups	15.86	*Φ*_CT_: 0.15	*ns*
	Among populations within groups	44.92	*Φ*_SC_: 0.53	< 0.0001
	Within populations	39.22	*Φ*_ST_: 0.60	< 0.0001
	Total	100		
Without grouping a priori	Among populations	60.64	*Φ*_ST_: 0.60	< 0.0001
	Within populations	39.36		
	Total	100		

*ns* = not significant

### Ancestral area reconstruction

**Fig. 4:**
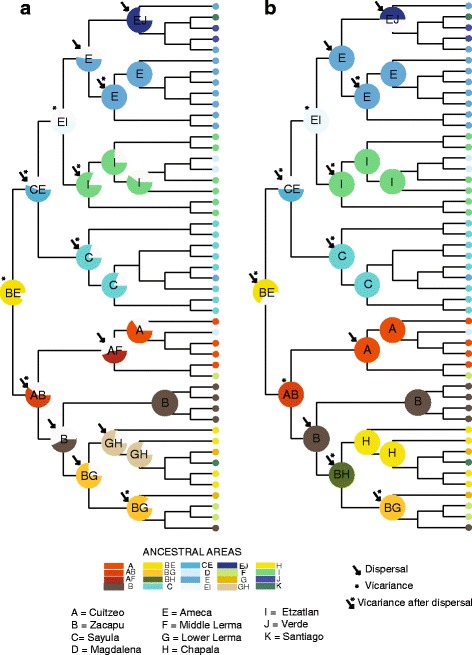
Ancestral area reconstruction with **a** DEC and **b** S-DIVA base in BEAST tree using the biogeographical regions proposed by Domínguez-Domínguez et al., (2006)

Ancestral area reconstruction using DEC and S-DIVA revealed a complex biogeographical history for *P. infans*, with several events of dispersal and vicariance (Figs. 3 and 4). Vicariance was most common in ancient events in comparison to dispersal events. For both analyses, the ancestral areas estimated for *P. infans* were Zacapu Lake and Ameca River, but with low probabilities (6.5% for DEC; 16.7% for S-DIVA). The same explanation for both analyses was found for the biogeographic history of the northwest populations of *P. infans* clustered in phylogenetic clade A. The biogeographical route for this clade included a dispersal event from the Ameca River toward the Sayula and Etzatlan-San Marcos regions, followed by a vicariant event that separated the populations of Ameca River with respect to Etzatlan-San Marcos and Sayula regions. A more recent dispersal event from the Ameca to the Verde River was recovered (S-DIVA > 50%, DEC > 27% of probabilities).

The biogeographical history of the southeastern clade B differs slightly in both biogeographical methods. For both methods, one dispersal event occurred from Zacapu toward Cuitzeo, followed by a vicariant event that separated these two regions. For DEC, dispersal events occurred from Zacapu toward the Lower Lerma and from here to Chapala, while there was also a recent dispersal event from Cuitzeo to the Middle Lerma. The S-DIVA differed with the DEC in that dispersal events from Zacapu were toward Chapala and once both regions were separated, a second dispersal event occurred from Chapala to the Lower Lerma (S-DIVA 100% and DEC > 60%) (Figs. 3 and 4).

### Historical demography

**Fig. 5:**
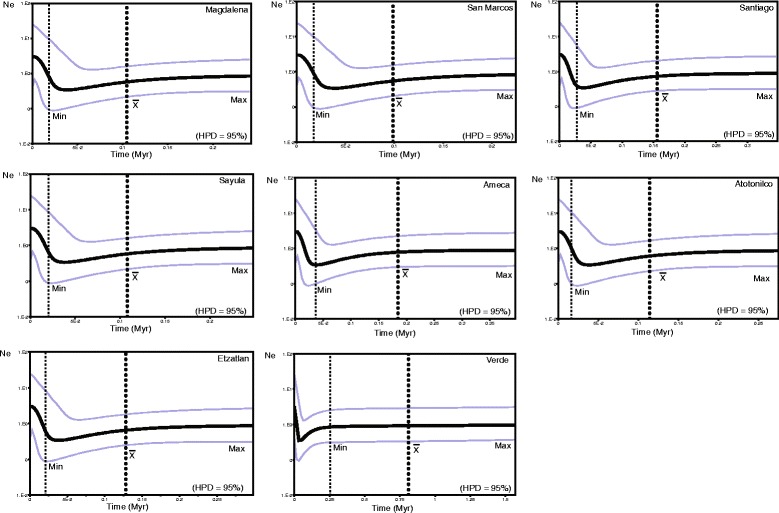
Demographic history of populations of each biogeographical region grouped in clade A using Bayesian Skyline Plots (BSP) from *cytb* sequences. Dotted lines represent the location of the upper bound (Max), the mean (X) and lower bound (Min) of the HPD = 95%

The BSP analyses for *cytb* for populations of clade A showed a demographic decline for Magdalena Lake, San Marcos, Sayula and Atotonilco Lakes (belonging to Sayula region), Ameca, Grande de Santiago and Etzatlan-San Marcos regions between 0.15 and 0.1 Myrs. More recently, after a demographic decline, a population expansion was detected in the last 0.025 Myr. For the Verde River, a population reduction was detected following a more recent population expansion. For this clade, the regions Zapotlan and Tamazula were not included due to the low number of individuals (Fig. 5).

**Fig. 6:**
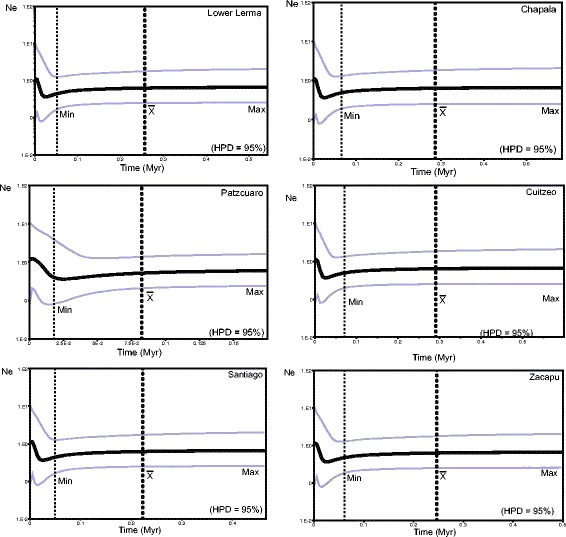
Demographic history of populations of each biogeographical region grouped in clade B using Bayesian Skyline Plots (BSP) from *cytb* sequences. Dotted lines represent the location of the upper bound (Max), the mean (X) and lower bound (Min) of the HPD = 95%

For clade B, all analyzed groups revealed a demographic decline in the last 0.15–0.1 Myr, followed by population expansion around ≤0.18 Myr, as found for clade A. For this clade the Balsas, Cotija and Middle Lerma basins were not included due the low number of samples (Fig. 6).

### *Poeciliopsis infans* Distribution modelling

**Fig. 7:**
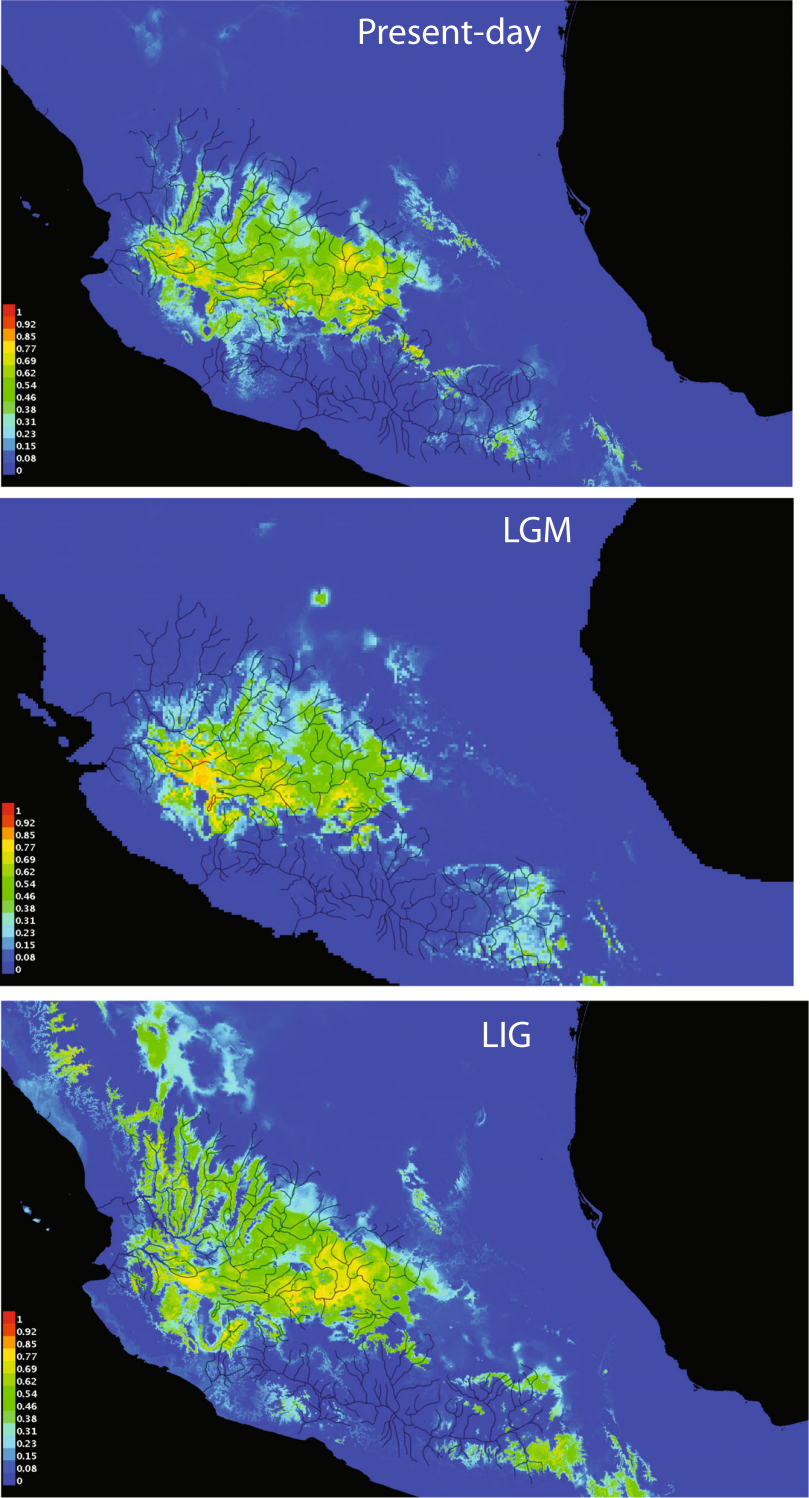
Species distribution modelling for: Current period, Last Inter Glacial (LIG: 0.15–0.10 Myr) and Last Glacial Maximum (LGM: 0.025–0.020 Myr), the probability of presence of the species is in scale of colors

The habitat suitability modelling for populations of *P. infans* estimated for current (1965–1978) and past (LIG: 0.15–0.10 Myr, and LGM: 0.025–0.020 Myr) time periods, showed high precision and acceptable predictive power with all models (AUC > 0.96) [69]. For the current conditions, the two variables with the highest gain were BIO3 Isothermality (BIO2/BIO7)*100 and BIO6 Min temperature of coldest month. In the LIG model, the two variables with the highest gain were BIO1 Annual Mean Temperature and BIO16 Precipitation of Wettest Quarter. For the LGM model, the two variables with the highest gain were BIO4 Temperature seasonality (standard deviation*100), and BIO6 Min Temperature of coldest month. The modelling of habitat suitability showed that in the LGM, the habitat suitability for *P. infans* was better in the lowlands, whereas for the LIG, the habitat suitability increased in the highlands (Fig. 7).

## Discussion

### Biogeographic and evolutionary history of *P. infans*

The recovery of two well-differentiated clades within *P. infans* (Fig 2 and Additional file 8) indicates a long history of isolation, with subsequent genetic differentiation, which seems to be linked to the intense volcanic and tectonic activity in central Mexico during the Pliocene (Figs. 2 and 3). This pattern previously has been reported, in which the genetic differentiation has been linked with the past configuration of the rivers more than the current hydrology of this region of central Mexico, as is the case for the goodeid, *Zoogoneticus quitzeoensis* (Bean, 1898), and for species of the cyprinid genus *Algansea* [30, 33].

Populations of *P. infans* are largely differentiated by the isolation of watersheds as have occurred in other freshwater fishes with low dispersal ability; however, some geographical features within a river or basin have been shown to be sufficient to differentiate populations of *P. infans* [26].

We recovered the samples from the Rio Grande de Santiago Basin grouped in the two main clades for phylogenetic analyses and in the two haplogroups for haplotype networks (A, B). This pattern, in which the samples from the Santiago River were grouped in two clades, previously was found in another study [26]. Specimens from the Rio Grande de Santiago Basin sampled upstream of the falls, el Salto de Juanacatlan, belong to clade B, whereas populations sampled downstream of the falls were grouped within clade A, suggesting that the Salto de Juanacatlan formation, a waterfall 20 m high, has been an important and ancient barrier promoting differentiation between populations. This geologic feature previously has been shown to represent a barrier for fish faunal interchange between the Santiago and Chapala Lake [26, 38].

Ancestral area reconstructions recovered very low marginal probabilities for biogeographical routes from the ancestral areas of all populations of *P. infans* (DEC = 0.01; S-DIVA = 0.08; Figs. 3 and 4). Therefore, both ancestral area reconstructions were unable to resolve the state of the node separating clade A from clade B, but were able to reconstruct the ancestral areas and biogeographical routes within each clade.

Despite these low marginal probabilities, the most plausible event that separated the Ameca River and Lake Zacapu of its ancestral area of distribution was a vicariant event. Our date estimation for the diversification of the two main clades (A and B) was ca. 2.83 Myr (1.25–4.41 Myr), between the middle Pliocene and the early Pleistocene periods (Figs. 3 and 4).

The geological activity during the middle Pliocene and early Pleistocene in central Mexico promoted the cladogenesis of clade A from clade B. This deep divergence of the two clades is coincident with the interruption of the ancient connection of the upper Ameca River with drainages in central Mexico by tectonic and volcanic activity at ca. 3–1 Myr [70], (Fig. 3). As a result, this dispersal route could have been blocked at the end of the Pliocene and during the Pleistocene. This occurred when the hydrological systems that shaped the complex Chapala-Lerma Paleosystem (Ameca River, Magdalena, Chapala, Lerma River and the lakes distributed along the Colima graben) became isolated due to volcanic and tectonic activity in the triple junction area, the Ameca and San Marcos faults at ca. 3.5–1.5 Myr (Fig. 3) [71].

The isolation of the region where clade A is distributed during the Pliocene, has also been reported in other freshwater species including the cyprinids *Yuriria amatlana* Domínguez-Domínguez et al., 2007 and *Algansea amecae* Pérez-Rodríguez et al., 2009, as well as the goodeid *Allotoca goslinei* Smith & Miller, 1987 [29, 30, 72, 73]. In addition, other freshwater organisms shown a similar pattern, as *Cambarellus chapalanus* (Faxon, 1898), which has two divergent genetic groups, one distributed in Chapala Lake and the other in the Ameca River Basin separated at ca. 2.6 Myr [32]. A similar pattern was mentioned before for *Poeciliopsis* [26], who suggested that the ancestors of the strictly northern clade of *Poeciliopsis* must have been distributed across the region presently occupied by our clade B, which includes Zacapu.

The discrepancies between the mitochondrial and nuclear genes are related to the mixture of some individuals of haplogroup A with haplogroup B. In this case, considering the high genetic distances with mitochondrial genes, the divergence times, and the results from the AMOVAs, we suggested that it could be the result of retention of ancestral polymorphisms, as has been shown for other freshwater fishes of central Mexico [74].

### Biogeography within clade A

The biogeographic analyses showed a dispersal event between Sayula and the Ameca basin, suggesting an early connection more than a million years ago. This connection was previously found for *P. infans* between the Ameca River and Atotonilco Lake of the Sayula region [26].

The isolation of the Ameca, Etzatlan-San Marcos, Magdalena and Lakes of the Sayula region, could be due to the formation of the current watersheds during the Pleistocene epoch ca. 0.95 Myr (95% HPD: 0.39–1.5 Myr; Figs. 2 and 3), when the connections of the Ameca River and Atotonilco-Sayula Lakes were disrupted by Pleistocene volcanism and the intense tectonic activity of the so called triple junction [71, 75]. This is also congruent with the presence of *Ameca splendens* Miller & Fitzsimons, 1971, in the Ameca River and Sayula regions, with a divergence time between the two populations calculated in less than a million of years [29].

Other dispersal events from the Ameca to Etzatlan-San Marcos and Magdalena regions were recovered. The climatic changes during this pluvial-interpluvial period, beginning ca. 0.90 Myr [4, 76], could have promoted this dispersal event when Magdalena Lake was considerably larger and extended to Etzatlan-San Marcos area [77]. After that time, the Ameca was isolated from the Etzatlan-San Marcos and Magdalena regions by a vicariant event ca. 0.72 Myr (DEC = 0.209; S-DIVA = 1.0), which could be associated with the reactivation of the Plan de Barrancas fault during the Quaternary (ca. 1.0 Myr), and with the Amatlán de Cañas half graben (Fig. 3) that was formed ca. 3.4 Myr [13, 70, 78].

Finally, a dispersal event from the Ameca River to the Verde and Santiago Rivers also was found (ca. 0.14 Myr), and this is supported by previous findings suggesting that these populations have been connected until very recently through stream capture of the Ameca and Verde Rivers, which was facilitated by the volcanism in the Tepic-Zacoalco graben [26, 79], (Fig. 3).

The biogeographic events that isolated the three recovered sub-clades are supported by the AMOVA analysis, maximizing the *Φ*_CT_ when samples were grouped into four groups, included the three sub-clades within clade A (Tables 5 and 6), but not when they were grouped according to biogeographic regions as have been proposed for other freshwater fishes, including goodeids and cyprinids [33, 36].

Since goodeids, cyprinids, and *P. infans* have evolved in spatiotemporal congruence, the differences found in the evolutionary history of *P. infans* could be related to the biogeographic origin of each group. *Poeciliopsis infans* has a Neotropical origin, whereas goodeids and cyprinids are of Neartic origin [18]. As a result, we expect that Quaternary climate changes have influenced genetic variation and the distributional patterns of *P. infans.* Moreover, the genetic diversity was higher for regions clustered in the clade A (lowlands) than for regions grouped in clade B (highlands)*.* This could also be linked to more stable high temperatures in lowland areas, which could also promote the diversification of populations found for this lowland clade (clade A). This pattern has been reported in plants that shown that the habitat and environment changes affect the genetic diversity [80–83], as is the case of *Caragana microphylla*, a species distributed in two different habitats that shown that populations from the high temperature region had lower genetic diversity than those from medium and low temperature regions [81]. The only exception of this pattern is the Magdalena Lake population, which has the lowest genetic diversity within this clade, a pattern that is explained due to the history of instability of this hydrological basin, with extreme and intermittent periods of flooding and drying [84], promoting recurrent events of bottlenecks and loss of genetic diversity.

Also, other factors, in addition to the environment, such as life-history traits, breeding systems, dispersal mechanism, geographic variation, range and life span and the histories of populations have affected genetic variation between populations [81–83].

### Biogeography within clade B

Our results are in accordance with the proposed connections and isolation between the Cuitzeo and Zacapu regions, which has occurred several times during the Pleistocene [85]. The connection between both regions has been postulated to occur through the Chucandiro-Huaniqueo paleo-river, a connection disrupted less than 1 Myr, due to the volcanism of the Tarasco corridor and the activity of the Northeast-Southeast fault system ca. 0.7 to 0.5 Myr [86], (Fig. 3). This disruption has been proposed as the cause of the isolation of different fish species between Cuitzeo and Zacapu including the goodeids *Skiffia lermae* (Meek, 1902), *Goodea atripinnis* (Jordan, 1880), *Alloophorus robustus* (Bean, 1892) and *Hubbsina turneri* (de Buen, 1940) [29, 85], and the cyprinids *Algansea tincella* (Valenciennes, 1844) and *Yuriria alta* (Jordan, 1880) [30, 72].

After the isolation of Zacapu and Cuitzeo, the DEC and S-DIVA showed a dispersal event from Cuitzeo towards the middle Lerma River, an event that is in accordance with recent isolation of this hydrological basin with respect to Zacapu and Cuitzeo lakes. This event was previously proposed for the goodeids *Hubbsina turneri* [85], and *Zoogoneticus quitzeoensis* (Bean, 1898)*,* that are distributed in Cuitzeo, Zacapu, and the middle Lerma [33], as well as *Neotoca bilineata* (Bean, 1887) for which a low level of genetic differentiation (mtDNA) was found for populations from Cuitzeo and the middle Lerma [87].

Regarding the second biogeographic route for this clade, which is different for the DEC and S-DIVA analyses, the most plausible route is a dispersal event from Zacapu toward the lower Lerma Basin, followed by a dispersal event toward Chapala Lake and the Santiago River respectively, as is found in DEC. Zacapu is currently connected with the lower Lerma through the Angulo River, for which *P. infans* could have dispersed toward Chapala Lake and the Santiago River via the lower Lerma. This information is congruent with the presence of *Notropis calientis* (Jordan & Snyder, 1899), *Yuriria alta*, *Algansea tincella*, *Xenotoca variata* (Bean, 1887), *Chapalichthys encaustus* (Jordan & Snyder, 1899), *Allotoca dugesii* (Bean, 1887) and *Moxostoma austrinum* Bean, 1880, in the Middle Lerma, Lower Lerma and Grande de Santiago basins, as well as in Chapala Lake region [35, 36].

### Human mediated dispersion

In general, we found biogeographic correspondence in the distribution of clades, but incongruences were also found for some populations and areas, as is the case of Balsas, Cotija and Patzcuaro, populations that shown a null genetic and haplotype diversities, with all sampled individuals belong to the most common haplotype of clade B.

These results have two possible explanations: 1) a secondary dispersal and colonization event, which is unlikely due to the historical isolation of all of those drainages with respect to contiguous drainages [29, 88], or 2) a founder effect due a dispersal event mediated by humans. Human-based introductions represent the most probable explanation according to the geographic distribution of related haplotypes and the null genetic diversity of Balsas, Cotija and Patzcuaro regions, since for Patzcuaro this species has been reported as a human-mediate introduction [89, 90] (Tables 3 and 4).

Several species in the family Poeciliidae have been introduced for mosquito control worldwide and have spread successfully to over 40 countries [91]. Other possible ways of introduction of poeciliids is the release of the organisms by aquarists, through the use of this fish as food source for commercial introduced fish, or accidentally transported with commercially important fishes stocked into water bodies, such a species of tilapia (*Oreochromis* and *Tilapia*), which have been widely introduced throughout Mexico [89, 92].

### Historical demography and distribution modeling

Fluctuations in population size shown by BSP in populations of both clades of *P. infans* agree with the continuous fluctuations of the climate and water levels of hydrological systems in central Mexico due to glacial and interglacial cycles [93].

For clade A, the analysis of historical demography showed a demographic decline at *ca* 0.15–0.1 Myr, followed by a recent population expansion, estimated to start around 0.025 Myr in most of the populations analyzed (Fig. 5). These genetic based analyses are congruent with the distribution modeling results, in which drainages where clade A is distributed, show restricted areas with high probabilities (≥0.77) to support populations of *P. infans* during the LIG (0.15–0.10 Myr), localized mainly to a small area within the Ameca region (Fig. 7). Whereas for the LGM (0.025–0.020 Myr), an increase in the areas with high probabilities (≥0.77) to support populations of *P. infans* is observed, covering most of the present day distribution of clade A populations. This recent population expansion for almost all populations of clade A could explain why the genetic diversity is highest in this clade rather than in the clade B. It is well known that stable populations that persisted from the LGM to the present harbor disproportionately large amounts of unique genetic diversity [94].

The decline in the clade B population seems to starts after 0.075 Myr, followed by a population expansion at ca. ≤0.018 Myr in all the populations analyzed, however, we take with caution the population expansion of some biogeographic regions for both clades, because the size increase is out of the HPD limits (Fig. 6).

These results are also congruent with the distribution modeling results, since the distribution modeling during LIG (0.15–0.10 Myr) also showed a high proportion of areas where clade B is distributed with high probabilities (≥0.77) to support populations of *P. infans*. Whereas for the LGM (0.025–0.020 Myr), a decrease in the area with high probability of presence (≥ 0.77) is observed for areas were clade B is distributed. These results are expected for a species with tropical preferences inhabiting highlands, where temperatures declines of 8.5 °C during LGM have been postulated [95], followed by a expansion of the distribution range when the last ice age ended and an increase of the temperature and water level of hydrological systems have been recorded [93]. It has been shown that the climatic fluctuations were accompanied by a loss of genetic diversity and even extinction of populations that were unable to adapt to these changes and find suitable conditions [96], as could be the case for *P. infans* that is restricted to basin drainages. This is congruent with the distribution modeling of clade B distributed in highlands of central Mexico, and explains the low genetic diversity found in almost all populations within this clade.

Finally, in the present day modeling that represents an Inter-glacial period, it showed an extended distributional area with a probability of presence ≥0.77 in the upper parts of the distributional range for both clades A and B. These results of population expansion in lower areas during Glacial Maximum and in upper areas during Inter Glacial periods are also congruent with the Neotropical origin of *P. infans*, suggesting that areas that maintain high temperatures are more suitable for *P. infans*. This is also congruent with reports suggesting the displacement of plant communities as a response to the climate cooling, resulting in the migration to low altitudes [97], for which the climate change in glacial cycles could be responsible of the modifications in the composition of the communities favoring more resistant species to the new environmental conditions [93, 97].

### Conservation implications

The regions occupied by *P. infans* have been heavily impacted by habitat loss due to overexploitation, pollution, habitat degradation, and the introduction of non-native species [98–100]. The negative impacts of these anthropogenic activities on native freshwater fishes of central Mexico are well known [33, 101–103]. These activities can exacerbate the loss of genetic diversity, which is especially harmful for a species with a long history of instability due to the natural climatic fluctuations, as is the case of *P. infans*. This is especially true for those populations distributed in clade B (Tables 3 and 4). For this reason, the establishment of each recovered clade and sub-clade as Operational Conservation Unit (OCU) [104] is necessary in order to conserve the unique genetic pool that each clade represents.

## Conclusions

The results of this study indicate that *P. infans* has had a long history of isolation and subsequent genetic differentiation, which appears to be linked to the intense volcanic and tectonic activity in central Mexico. The separation of the two recovered clades appears to have been promoted by the geological activity during the middle Pliocene, and early Pleistocene in central Mexico, *ca.* 2.83 Myr. *Poeciliopsis infans* is a co-distributed species with other group of fishes as goodeids and cyprinids, evolving in spatiotemporal congruence. The differences in the recovered biogeographic patterns of *P. infans* is likely related to the biogeographic origin of each group, since *P. infans* has Neotropical origin, and goodeids and cyprinids are of Neartic origin. Populations of *P. infans* distributed in lowlands showed a higher level of genetic diversity than populations distributed in highlands, which could be linked to more stable and higher temperatures in lowland areas. Finally, fluctuations in population size are supported by the continuous fluctuations of the climate and water levels of hydrological systems in central Mexico due to glacial and interglacial cycles.

## Additional files

Additional file 1: Tissue voucher number, and access number of GenBank. (DOC 416 kb)

Additional file 2: Primers, PCR conditions, and References. (DOC 35 kb)

Additional file 3: Models selected with Akaike information criterion and the parameters of each gene. (DOC 32 kb)

Additional file 4: Geographical coordinates of the 162 sites registers in Colección de Peces de la Universidad Michoacana de San Nicolás de Hidalgo used as presence data for species distribution modelling. (DOC 161 kb)

Additional file 5: Ambiguously Aligned Regions for *S7*. (DOC 49 kb)

Additional file 6: The Bayesian inference tree of *P. infans* from *cytb* mitochondrial gene (1083 bp). Bayesian posterior probability (> 0.9; above the branches) and maximum likelihood bootstrap values (> 80%; below the branches) are indicated. (DOC 943 kb)

Additional file 7: The Bayesian inference tree of *P. infans* from *coxI* mitochondrial gene (631 bp). Bayesian posterior probability (> 0.9; above the branches) and maximum likelihood bootstrap values (> 80%; below the branches) are indicated. (DOC 548 kb)

Additional file 8: The Bayesian inference tree of *P. infans* from concatenated sequences of two mitochondrial genes (*cytb*, *coxI;* 1771 bp). Bayesian posterior probability (> 0.9; above the branches) and maximum likelihood bootstrap values (> 80%; below the branches) are indicated. (DOC 738 kb)

Additional file 9: The Bayesian inference tree of *P. infans* from *RHO* nuclear gene (845 bp). Bayesian posterior probability (> 0.9; above the branches) and maximum likelihood bootstrap values (> 80%; below the branches) are indicated. (DOC 469 kb)

Additional file 10: The Bayesian inference tree of *P. infans* from *S7* nuclear gene (859 bp). Bayesian posterior probability (> 0.9; above the branches) and maximum likelihood bootstrap values (> 80%; below the branches) are indicated. (DOC 715 kb)

Additional file 11: The Bayesian inference tree of *P. infans* from concatenated sequences of two nuclear genes (*S7* and *RHO:* 1704 bp). Bayesian posterior probability (> 0.9) and maximum likelihood bootstrap values (> 80%) are indicated. (DOC 2382 kb)

Additional file 12: Haplotype networks for nuclear genes, a) *S7* gene, b) *RHO* gene. The two recovered haplogroups are show with labels A and B. (DOC 1189 kb)

## Abbreviations

mtDNA: Mitochondrial Deoxyribonucleic Acid
nDNA: Nuclear Deoxyribonucleic Acid;
SEMARNAT: Secretaria de Medio Ambiente y Recursos Naturales
