# Supplementary material for: Evolving in the highlands: the case of the Neotropical Lerma live-bearing Poeciliopsis infans (Woolman, 1894) (Cyprinodontiformes: Poeciliidae) in Central Mexico
Source: BMC Evol Biol. 2018 Apr 20;18:56. doi: 10.1186/s12862-018-1172-7 (PMC5910627; doi:10.1186/s12862-018-1172-7)
Supplement: Supplementary file 1 — Tissue voucher number, and access number of GenBank. (DOC 416 kb) [file 12862_2018_1172_MOESM1_ESM.doc]

Additional file 1. Tissue voucher number, and access number of GenBank

| Site | Locality | Biogeographic region | Voucher number | Access number of GenBank  *Cytb* *coxI*  *RHO* *S7* | | | |  | | | | | | | | | | | | |
| --- | --- | --- | --- | --- | --- | --- | --- | --- | --- | --- | --- | --- | --- | --- | --- | --- | --- | --- | --- | --- |
| 1 | Los Venados | Magdalena | 9429 | MG028024 | MG028296 | MG100624 | MG366198 |  | | | | | | | | | | | | |
| 1 |  |  | 9429B | --- | --- | NO | MG366199 |  | | | | | | | | | | | | |
| 1 |  |  | 9431 | MG028025 | MG028297 | MG100625 | MG366200 |  | | | | | | | | | | | | |
| 1 |  |  | 9431B | --- | --- | MG100626 | MG366201 |  | | | | | | | | | | | | |
| 1 |  |  | 9432 | MG028026 | MG028298 | NO | NO |  | | | | | | | | | | | | |
| 1 |  |  | 9434 | MG028027 | MG028299 | MG100627 | NO |  | | | | | | | | | | | | |
| 1 |  |  | 9434B | --- | --- | MG100628 | NO |  | | | | | | | | | | | | |
| 1 |  |  | 9435 | MG028028 | MG028300 | MG100629 | MG366202 |  | | | | | | | | | | | | |
| 2 | Laguna | Magdalena | 4600 | MG028023 | MG028295 | MG100622 | MG366196 |  | |  | | |  | | |  | |  | |  |
| 2 |  |  | 4600B | --- | --- | MG100623 | MG366197 |  | |  | | |  | | |  | |  | |  |
| 2 |  |  | 9959 | MG028029 | MG028301 | MG100630 | MG366203 |  | | | | | | | | | | | | |
| 2 |  |  | 11695 | MG028030 | MG028302 | NO | MG366204 |  | | | | | | | | | | | | |
| 2 |  |  | 11696 | MG028031 | NO | NO | NO |  | | | | | | | | | | | | |
| 2 |  |  | 11697 | MG028032 | MG028303 | NO | MG366205 |  | | | | | | | | | | | | |
| 2 |  |  | 11697B | --- | --- | NO | MG366206 |  | | | | | | | | | | | | |
| 2 |  |  | 11698 | MG028033 | MG028304 | MG100631 | MG366207 |  | | | | | | | | | | | | |
| 2 |  |  | 11699 | MG028034 | MG028305 | MG100632 | MG366208 |  | | | | | | | | | | | | |
| 2 |  |  | 11699B | --- | ---- | NO | MG366209 |  | | | | | | | | | | | | |
| 2 |  |  | 11700 | MG028035 | MG028306 | MG100633 | MG366210 |  | | | | | | | | | | | | |
| 2 |  |  | 28074 | MG028036 | NO | NO | MG366211 |  | | | | | | | | | | | | |
| 2 |  |  | 28074B | --- | --- | NO | MG366212 |  | | | | | | | | | | | | |
| 2 |  |  | 28075 | MG028037 | MG028307 | NO | MG366213 |  | | | | | | | | | | | | |
| 2 |  |  | 28076 | MG028038 | NO | MG100634 | MG366214 |  | | | | | | | | | | | | |
| 3 | Presa San Ignacio | Ameca | 4632 | MG028039 | MG028308 | MG100635 | MG366215 |  | |  | | |  | | |  | |  | |  |
| 3 |  |  | 4632B | --- | --- | NO | MG366216 |  | |  | | |  | | |  | |  | |  |
| 3 |  |  | 42907 | MG028070 | MG028334 | MG100663 | NO |  | | | | | | | | | | | | |
| 3 |  |  | 42908 | MG028071 | MG028335 | NO | NO |  | | | | | | | | | | | | |
| 3 |  |  | 42909 | MG028072 | MG028336 | MG100664 | NO |  | | | | | | | | | | | | |
| 3 |  |  | 42910 | MG028073 | MG028337 | MG100665 | NO |  | | | | | | | | | | | | |
| 3 |  |  | 42911 | MG028074 | NO | MG100666 | MG366254 |  | | | | | | | | | | | | |
| 3 |  |  | 42927 | MG028075 | MG028338 | MG100667 | MG366255 |  | | | | | | | | | | | | |
| 4 | Chapulimita | Ameca | 4654 | MG028040 | MG028309 | MG100636 | MG366217 |  | | | | | | | | | | | | |
| 4 |  |  | 4654B | --- | --- | MG100637 | MG366218 |  | | | | | | | | | | | | |
| 4 |  |  | 4655 | MG028041 | MG028310 | MG100638 | MG366219 |  | | | | | | | | | | | | |
| 4 |  |  | 4655B | --- | --- | NO | MG366220 |  | | | | | | | | | | | | |
| 5 | Salida presa Tecuan | Ameca | 5049 | MG028042 | MG028311 | MG100639 | MG366221 |  | | | | | | | | | | | | |
| 5 |  |  | 5049B | --- | --- | NO | MG366222 |  | | | | | | | | | | | | |
| 5 |  |  | 5050 | MG028043 | NO | MG100640 | MG366223 |  | | | | | | | | | | | | |
| 5 |  |  | 5051 | MG028044 | MG028312 | MG100641 | MG366224 |  | | | | | | | | | | | | |
| 5 |  |  | 5051B | --- | --- | NO | MG366225 |  | | | | | | | | | | | | |
| 5 |  |  | 5052 | MG028045 | MG028313 | MG100642 | MG366226 |  | | | | | | | | | | | | |
| 5 |  |  | 5052B | --- | --- | NO | MG366227 |  | | | | | | | | | | | | |
| 5 |  |  | 5053 | MG028046 | MG028314 | NO | NO |  | | | | | | | | | | | | |
| 5 |  |  | 5054 | MG028047 | MG028315 | MG100643 | MG366228 |  | | | | | | | | | | | | |
| 5 |  |  | 5054B | --- | --- | NO | MG366229 |  | | | | | | | | | | | | |
| 5 |  |  | 5059 | MG028048 | MG028317 | NO | NO |  | | | | | | | | | | | | |
| 6 | Manantial Los Veneros | Ameca | 9920 | MG028049 | MG028318 | MG100644 | MG366230 |  | | | | | | | | | | | | |
| 6 |  |  | 9920B | --- | --- | NO | MG366231 |  | | | | | | | | | | | | |
| 6 |  |  | 9921 | MG028050 | MG028319 | MG100645 | MG366232 |  | | | | | | | | | | | | |
| 7 | Tala, Río Salado | Ameca | 11841 | MG028051 | MG028320 | MG100646 | MG366233 |  | | | | | | | | | | | | |
| 7 |  |  | 11842 | MG028052 | MG028321 | MG100647 | NO |  | | | | | | | | | | | | |
| 7 |  |  | 11843 | MG028053 | MG028322 | MG100648 | NO |  | | | | | | | | | | | | |
| 8 | Amatlán de cañas | Ameca | 11979 | MG028054 | MG028323 | MG100649 | MG366234 |  | | | | | | | | | | | | |
| 8 |  |  | 11979B | --- | --- | NO | MG366235 |  | | | | | | | | | | | | |
| 8 |  |  | 11980 | MG028055 | MG028324 | MG100650 | MG366236 |  | | | | | | | | | | | | |
| 8 |  |  | 11980B | --- | --- | MG100651 | MG366237 |  | | | | | | | | | | | | |
| 8 |  |  | 11981 | MG028056 | NO | MG100652 | MG366238 |  | | | | | | | | | | | | |
| 8 |  |  | 11981B | --- | --- | MG100653 | MG366239 |  | | | | | | | | | | | | |
| 8 |  |  | 11982 | MG028057 | MG028325 | NO | MG366240 |  | | | | | | | | | | | | |
| 8 |  |  | 11982B | --- | --- | NO | MG366241 |  | | | | | | | | | | | | |
| 8 |  |  | 11984 | MG028058 | MG028326 | MG100654 | MG366242 |  | | | | | | | | | | | | |
| 8 |  |  | 11985 | MG028059 | MG028327 | MG100655 | MG366243 |  | | | | | | | | | | | | |
| 8 |  |  | 11985B | --- | --- | NO | MG366244 |  | | | | | | | | | | | | |
| 9 | Teuchitlán | Ameca | 28085 | MG028060 | NO | NO | NO |  | | | | | | | | | | | | |
| 9 |  |  | 28086 | MG028061 | NO | NO | NO |  | | | | | | | | | | | | |
| 9 |  |  | 28087 | MG028062 | NO | NO | MG366245 |  | | | | | | | | | | | | |
| 9 |  |  | 28088 | MG028063 | NO | NO | NO |  | | | | | | | | | | | | |
| 9 |  |  | 28089 | MG028064 | MG028328 | MG100656 | MG366246 |  | | | | | | | | | | | | |
| 9 |  |  | 28089B | --- | --- | NO | MG366247 |  | | | | | | | | | | | | |
| 9 |  |  | 31540 | MG028065 | MG028329 | MG100657 | MG366248 |  | | | | | | | | | | | | |
| 9 |  |  | 31540B | --- | --- | MG100658 | NO |  | | | | | | | | | | | | |
| 9 |  |  | 31541 | MG028066 | MG028330 | MG100659 | MG366249 |  | | | | | | | | | | | | |
| 9 |  |  | 31541B | --- | --- | MG100660 | MG366250 |  | | | | | | | | | | | | |
| 9 |  |  | 31542 | MG028067 | MG028331 | MG100661 | MG366251 |  | | | | | | | | | | | | |
| 9 |  |  | 31543 | MG028068 | MG028332 | MG100662 | MG366252 |  | | | | | | | | | | | | |
| 9 |  |  | 31543B | --- | --- | NO | MG366253 |  | | | | | | | | | | | | |
| 9 |  |  | 31544 | MG028069 | MG028333 | NO | NO |  | | | | | | | | | | | | |
| 10 | S. M. San Julian | Verde | 4672 | MG028224 | MG028487 | NO | MG366429 |  | | | | | | | | | | | | |
| 10 |  |  | 4673 | MG028225 | MG028488 | MG100780 | NO |  | | | | | | | | | | | | |
| 10 |  |  | 4674 | MG028226 | MG028489 | MG100781 | MG366430 |  | | | | | | | | | | | | |
| 10 |  |  | 4674B | --- | --- | MG100782 | NO |  | | | | | | | | | | | | |
| 11 | San Nicolás | Verde | 35449 | NO | MG028490 | NO | NO |  | | | | | | | | | | | | |
| 11 |  |  | 35450 | MG028227 | MG028491 | MG100783 | NO |  | | | | | | | | | | | | |
| 11 |  |  | 35451 | MG028228 | MG028492 | MG100784 | NO |  | | | | | | | | | | | | |
| 11 |  |  | 35452 | NO | MG028493 | NO | NO |  | | | | | | | | | | | | |
| 11 |  |  | 35453 | MG028229 | MG028494 | MG100785 | MG366431 |  | | | | | | | | | | | | |
| 11 |  |  | 35454 | MG028230 | MG028495 | MG100786 | NO |  | | | | | | | | | | | | |
| 11 |  |  | 35455 | MG028231 | MG028496 | MG100787 | NO |  | | | | | | | | | | | | |
| 11 |  |  | 35456 | MG028232 | MG028497 | MG100788 | MG366432 |  | | | | | | | | | | | | |
| 11 |  |  | 35456B | --- | --- | NO | MG366433 |  | | | | | | | | | | | | |
| 11 |  |  | 35457 | MG028233 | MG028498 | MG100789 | MG366434 |  | | | | | | | | | | | | |
| 11 |  |  | 35457B | --- | --- | NO | MG366435 |  | | | | | | | | | | | | |
| 11 |  |  | 35458 | MG028234 | MG028499 | NO | NO |  | | | | | | | | | | | | |
| 11 |  |  | 35459 | MG028235 | MG028500 | NO | NO |  | | | | | | | | | | | | |
| 11 |  |  | 36260 | MG028242 | MG028507 | MG100796 | MG366437 |  | | | | | | | | | | | | |
| 11 |  |  | 36260B | --- | --- | NO | MG366438 |  | | | | | | | | | | | | |
| 11 |  |  | 36261 | MG028243 | MG028508 | MG100797 | MG366439 |  | | | | | | | | | | | | |
| 11 |  |  | 36261B | --- | --- | NO | MG366440 |  | | | | | | | | | | | | |
| 12 | Arroyo La Estancia | Verde | 35460 | MG028236 | MG028501 | MG100790 | MG366436 |  | | | | | | | | | | | | |
| 12 |  |  | 35461 | MG028237 | MG028502 | MG100791 | NO |  | | | | | | | | | | | | |
| 13 | Río Colorado | Verde | 36256 | MG028238 | MG028503 | MG100792 | NO |  | | | | | | | | | | | | |
| 13 |  |  | 36257 | --- | --- | MG100793 | NO |  | | | | | | | | | | | | |
| 13 |  |  | 36258 | MG028240 | MG028505 | MG100794 | NO |  | | | | | | | | | | | | |
| 13 |  |  | 36259 | MG028241 | MG028506 | MG100795 | NO |  | | | | | | | | | | | | |
| 14 | Río Xoconostle-San Juan | Medio Lerma | 4948 | MG028076 | MG028339 | MG100668 | YES |  | | | | | | | | | | | | |
| 14 |  |  | 4948B | --- | --- | MG100669 | MG366256 |  | | | | | | | | | | | | |
| 14 |  |  | 4949 | MG028077 | MG028340 | MG100670 | MG366257 |  | | | | | | | | | | | | |
| 14 |  |  | 12810 | MG028078 | MG028341 | MG100671 | MG366258 |  | | | | | | | | | | | | |
| 15 | Manantial Andrés-Figueroa | Sayula | 5023 | MG028079 | MG028342 | NO | MG366259 |  | | | | | | | | | | | | |
| 15 |  |  | 5024 | NO | MG028343 | NO | MG366260 |  | | | | | | | | | | | | |
| 15 |  |  | 5026 | MG028080 | MG028344 | MG100672 | MG366261 |  | | | | | | | | | | | | |
| 15 |  |  | 5026B | --- | --- | MG100673 | NO |  | | | | | | | | | | | | |
| 15 |  |  | 5027 | MG028081 | MG028345 | NO | MG366262 |  | | | | | | | | | | | | |
| 15 |  |  | 5044 | MG028082 | MG028346 | MG100674 | NO |  | | | | | | | | | | | | |
| 15 |  |  | 5044B | --- | --- | MG100675 | NO |  | | | | | | | | | | | | |
| 15 |  |  | 5047 | MG028083 | MG028347 | MG100676 | MG366263 |  | | | | | | | | | | | | |
| 15 |  |  | 18962 | MG028104 | MG028369 | MG100692 | MG366292 |  | | | | | | | | | | | | |
| 15 |  |  | 18963 | MG028105 | MG028370 | NO | NO |  | | | | | | | | | | | | |
| 15 |  |  | 18964 | MG028106 | MG028371 | MG100693 | MG366293 |  | | | | | | | | | | | | |
| 15 |  |  | 18964B | --- | --- | MG100694 | NO |  | | | | | | | | | | | | |
| 16 | Manantial San Marcos | Sayula | 28115 | MG028109 | MG028375 | MG100699 | MG366300 |  | | | | | | | | | | | | |
| 16 |  |  | 28116 | MG028110 | MG028376 | MG100700 | MG366301 |  | | | | | | | | | | | | |
| 16 |  |  | 28116B | --- | --- | MG100701 | NO |  | | | | | | | | | | | | |
| 16 |  |  | 28117 | MG028111 | MG028377 | MG100702 | MG366302 |  | | | | | | | | | | | | |
| 16 |  |  | 28118 | MG028112 | MG028378 | MG100703 | MG366303 |  | | | | | | | | | | | | |
| 16 |  |  | 28119 | MG028113 | MG028379 | MG100704 | MG366304 |  | | | | | | | | | | | | |
| 17 | Canal Presa Buena Vista | Sayula | 8360 | MG028085 | MG028349 | MG100678 | MG366266 |  | | | | | | | | | | | | |
| 17 |  |  | 8360B | --- | --- | NO | MG366267 |  | | | | | | | | | | | | |
| 17 |  |  | 8361 | NO | MG028350 | NO | NO |  | | | | | | | | | | | | |
| 17 |  |  | 8362 | MG028086 | MG028351 | NO | MG366268 |  | | | | | | | | | | | | |
| 17 |  |  | 8363 | MG028087 | MG028352 | NO | MG366269 |  | | | | | | | | | | | | |
| 17 |  |  | 8363B | --- | --- | NO | MG366270 |  | | | | | | | | | | | | |
| 17 |  |  | 8364 | MG028088 | MG028353 | MG100679 | MG366271 |  | | | | | | | | | | | | |
| 17 |  |  | 8364B | --- | --- | NO | MG366272 |  | | | | | | | | | | | | |
| 17 |  |  | 8365 | MG028089 | MG028354 | NO | MG366273 |  | | | | | | | | | | | | |
| 17 |  |  | 8366 | MG028090 | MG028355 | MG100680 | MG366274 |  | | | | | | | | | | | | |
| 17 |  |  | 8366B | --- | --- | NO | MG366275 |  | | | | | | | | | | | | |
| 18 | Villa corona | Sayula | 5070 | MG028084 | MG028348 | MG100677 | MG366264 |  | | | | | | | | | | | | |
| 18 |  |  | 5070B | --- | --- | NO | MG366265 |  | | | | | | | | | | | | |
| 18 |  |  | 28109 | MG028107 | MG028373 | MG100695 | MG366296 |  | | | | | | | | | | | | |
| 18 |  |  | 28109B | --- | --- | MG100696 | MG366297 |  | | | | | | | | | | | | |
| 18 |  |  | 28110 | MG028108 | MG028374 | MG100697 | MG366298 |  | | | | | | | | | | | | |
| 18 |  |  | 28110B | --- | --- | MG100698 | MG366299 |  | | | | | | | | | | | | |
| 19 | Manantial Cuyacapán | Sayula | 10565 | MG028091 | MG028356 | MG100681 | MG366276 |  | | | | | | | | | | | | |
| 19 |  |  | 10565B | --- | --- | NO | MG366277 |  | | | | | | | | | | | | |
| 19 |  |  | 10566 | MG028092 | MG028357 | MG100682 | MG366278 |  | | | | | | | | | | | | |
| 19 |  |  | 10566B | --- | --- | NO | MG366279 |  | | | | | | | | | | | | |
| 19 |  |  | 10567 | MG028093 | MG028358 | NO | MG366280 |  | | | | | | | | | | | | |
| 19 |  |  | 10567B | --- | --- | NO | MG366281 |  | | | | | | | | | | | | |
| 19 |  |  | 10568 | MG028094 | MG028359 | MG100683 | MG366282 |  | | | | | | | | | | | | |
| 19 |  |  | 10568B | --- | --- | NO | MG366283 |  | | | | | | | | | | | | |
| 19 |  |  | 10570 | MG028095 | MG028360 | MG100684 | MG366284 |  | | | | | | | | | | | | |
| 19 |  |  | 10571 | MG028096 | MG028361 | MG100685 | MG366285 |  | | | | | | | | | | | | |
| 19 |  |  | 10571B | --- | --- | NO | MG366286 |  | | | | | | | | | | | | |
| 19 |  |  | 10572 | MG028097 | MG028362 | MG100686 | NO |  | | | | | | | | | | | | |
| 19 |  |  | 10573 | MG028098 | MG028363 | MG100687 | MG366287 |  | | | | | | | | | | | | |
| 19 |  |  | 10573B | --- | --- | MG100688 | MG366288 |  | | | | | | | | | | | | |
| 19 |  |  | 10574 | MG028099 | MG028364 | NO | NO |  | | | | | | | | | | | | |
| 19 |  |  | 10575 | MG028100 | MG028365 | MG100689 | NO |  | | | | | | | | | | | | |
| 19 |  |  | 10577 | MG028101 | MG028366 | NO | NO |  | | | | | | | | | | | | |
| 19 |  |  | 10578 | MG028102 | MG028367 | MG100690 | MG366289 |  | | | | | | | | | | | | |
| 19 |  |  | 10578B | NO | NO | MG100691 | MG366290 |  | | | | | | | | | | | | |
| 19 |  |  | 42833 | MG028115 | MG028381 | NO | NO |  | | | | | | | | | | | | |
| 19 |  |  | 42834 | NO | MG028382 | NO | NO |  | | | | | | | | | | | | |
| 19 |  |  | 42835 | MG028116 | MG028383 | MG100705 | NO |  | | | | | | | | | | | | |
| 19 |  |  | 42836 | NO | MG028384 | NO | NO |  | | | | | | | | | | | | |
| 19 |  |  | 42932 | MG028117 | MG028385 | MG100706 | MG366305 |  | | | | | | | | | | | | |
| 20 | Laguna de Zapotlán | Sayula | 14095 | MG028103 | MG028368 | NO | MG366291 |  | | | | | | | | | | | | |
| 20 |  |  | 42814 | MG028114 | MG028380 | NO | NO |  | | | | | | | | | | | | |
| 21 | Río Las Puentes | Chapala | 17979 | NO | MG028456 | MG100749 | MG366382 |  | | | | | | | | | | | | |
| 21 |  |  | 17979B | --- | --- | MG100750 | NO |  | | | | | | | | | | | | |
| 21 |  |  | 17980 | NO | MG028457 | NO | NO |  | | | | | | | | | | | | |
| 21 |  |  | 17981 | MG028189 | MG028458 | NO | MG366383 |  | | | | | | | | | | | | |
| 21 |  |  | 17981B | --- | --- | NO | MG366384 |  | | | | | | | | | | | | |
| 21 |  |  | 17982 | MG028190 | MG028459 | MG100751 | MG366385 |  | | | | | | | | | | | | |
| 21 |  |  | 17982B | --- | --- | NO | MG366386 |  | | | | | | | | | | | | |
| 21 |  |  | 17983 | MG028191 | NO | MG100752 | MG366387 |  | | | | | | | | | | | | |
| 21 |  |  | 17984 | MG028192 | MG028460 | MG100753 | MG366388 |  | | | | | | | | | | | | |
| 21 |  |  | 17993 | MG028193 | MG028461 | MG100754 | MG366389 |  | | | | | | | | | | | | |
| 21 |  |  | 17993B | --- | --- | NO | MG366390 |  | | | | | | | | | | | | |
| 21 |  |  | 17995 | MG028194 | MG028462 | MG100755 | MG366391 |  | | | | | | | | | | | | |
| 21 |  |  | 17995B | --- | --- | NO | MG366392 |  | | | | | | | | | | | | |
| 22 | Cojumatlán | Chapala | 28130 | MG028195 | NO | NO | NO |  | | | | | | | | | | | | |
| 22 |  |  | 28131 | MG028196 | MG028463 | NO | NO |  | | | | | | | | | | | | |
| 22 |  |  | 28132 | MG028197 | MG028464 | MG100756 | NO |  | | | | | | | | | | | | |
| 22 |  |  | 28132B | --- | --- | MG100757 |  |  | | | | | | | | | | | | |
| 23 | Los Negritos | Chapala | 28186 | MG028198 | NO | NO | MG366393 |  | | | | | | | | | | | | |
| 23 |  |  | 28189 | MG028199 | NO | NO | MG366394 |  | | | | | | | | | | | | |
| 23 |  |  | 28189B | --- | --- | NO | MG366395 |  | | | | | | | | | | | | |
| 23 |  |  | 28191 | MG028200 | MG028465 | NO | MG366396 |  | | | | | | | | | | | | |
| 23 |  |  | 28192 | MG028201 | MG028466 | MG100758 | MG366397 |  | | | | | | | | | | | | |
| 23 |  |  | 28193 | MG028202 | MG028467 | MG100759 | MG366398 |  | | | | | | | | | | | | |
| 23 |  |  | 28193B | --- | --- | NO | MG366399 |  | | | | | | | | | | | | |
| 24 | Presa Nueva | Chapala | 28264 | MG028203 | MG028468 | MG100760 | MG366400 |  | | | | | | | | | | | | |
| 24 |  |  | 28264B | --- | --- | NO | MG366401 |  | | | | | | | | | | | | |
| 24 |  |  | 28265 | MG028204 | MG028469 | NO | MG366402 |  | | | | | | | | | | | | |
| 24 |  |  | 28265B | --- | --- | NO | MG366403 |  | | | | | | | | | | | | |
| 24 |  |  | 28266 | MG028205 | MG028470 | MG100761 | MG366404 |  | | | | | | | | | | | | |
| 24 |  |  | 28266 | --- | --- | NO | MG366405 |  | | | | | | | | | | | | |
| 24 |  |  | 28267 | MG028206 | MG028471 | MG100762 | MG366406 |  | | | | | | | | | | | | |
| 24 |  |  | 28267B | --- | --- | NO | MG366407 |  | | | | | | | | | | | | |
| 24 |  |  | 28268 | MG028207 | MG028472 | MG100763 | MG366408 |  | | | | | | | | | | | | |
| 24 |  |  | 28268B | --- | --- | NO | MG366409 |  | | | | | | | | | | | | |
| 25 | Manantial La Mintzita | Cuitzeo | 10153 | MG028118 | MG028386 | MG100707 | MG366306 |  | | | | | | | | | | | | |
| 25 |  |  | 10154 | MG028119 | MG028387 | MG100708 | MG366307 |  | | | | | | | | | | | | |
| 25 |  |  | 10154B | --- | ---- | NO | MG366308 |  | | | | | | | | | | | | |
| 25 |  |  | 10155 | MG028120 | MG028388 | NO | MG366309 |  | | | | | | | | | | | | |
| 25 |  |  | 10155B | --- | --- | NO | MG366310 |  | | | | | | | | | | | | |
| 25 |  |  | 10156 | MG028121 | MG028389 | NO | NO |  | | | | | | | | | | | | |
| 25 |  |  | 10157 | MG028122 | MG028390 | NO | MG366311 |  | | | | | | | | | | | | |
| 25 |  |  | 10164 | MG028123 | MG028391 | MG100709 | MG366312 |  | | | | | | | | | | | | |
| 25 |  |  | 10164B | --- | --- | NO | MG366313 |  | | | | | | | | | | | | |
| 26 | Ojo de Agua San Cristóbal | Cuitzeo | 10359 | MG028124 | MG028392 | NO | MG366314 |  | | | | | | | | | | | | |
| 26 |  |  | 10359B | --- | --- | NO | MG366315 |  | | | | | | | | | | | | |
| 26 |  |  | 10360 | MG028125 | MG028393 | MG100710 | NO |  | | | | | | | | | | | | |
| 26 |  |  | 10361 | MG028126 | MG028394 | MG100711 | NO |  | | | | | | | | | | | | |
| 26 |  |  | 10362 | MG028127 | MG028395 | MG100712 | NO |  | | | | | | | | | | | | |
| 26 |  |  | 10364 | MG028128 | MG028396 | MG100713 | MG366316 |  | | | | | | | | | | | | |
| 26 |  |  | 10365 | MG028129 | MG028397 | MG100714 | MG366317 |  | | | | | | | | | | | | |
| 26 |  |  | 10366 | NO | MG028398 | NO | MG366318 |  | | | | | | | | | | | | |
| 26 |  |  | 10369 | MG028130 | MG028399 | NO | MG366319 |  | | | | | | | | | | | | |
| 26 |  |  | 10369B | --- | --- | NO | MG366320 |  | | | | | | | | | | | | |
| 26 |  |  | 10370 | MG028131 | MG028400 | MG100715 | MG366321 |  | | | | | | | | | | | | |
| 26 |  |  | 10372 | MG028132 | MG028401 | NO | MG366322 |  | | | | | | | | | | | | |
| 27 | Embarcadero Principal | Patzcuaro | 10265 | MG028133 | MG028402 | NO | MG366323 |  | | | | | | | | | | | | |
| 27 |  |  | 10266 | MG028134 | NO | NO | MG366324 |  | | | | | | | | | | | | |
| 27 |  |  | 10267 | NO | MG028403 | NO | NO |  | | | | | | | | | | | | |
| 27 |  |  | 10286 | MG028135 | MG028404 | NO | MG366325 |  | | | | | | | | | | | | |
| 27 |  |  | 10287 | MG028136 | MG028405 | MG100716 | MG366326 |  | | | | | | | | | | | | |
| 2 |  |  | 10288 | MG028137 | MG028406 | NO | NO |  | | | | | | | | | | | | |
| 27 |  |  | 10289 | NO | MG028407 | NO | NO |  | | | | | | | | | | | | |
| 27 |  |  | 10290 | MG028138 | MG028408 | NO | MG366327 |  | | | | | | | | | | | | |
| 28 | Urandén | Patzcuaro | 23906 | MG028139 | MG028409 | MG100717 | MG366328 |  | | | | | | | | | | | | |
| 28 |  |  | 23906B | --- | --- | MG100718 |  |  | | | | | | | | | | | | |
| 28 |  |  | 26806 | MG028140 | MG028410 | MG100719 | MG366329 |  | | | | | | | | | | | | |
| 28 |  |  | 26807 | MG028141 | MG028411 | NO | NO |  | | | | | | | | | | | | |
| 28 |  |  | 26808 | MG028142 | MG028412 | MG100720 | MG366330 |  | | | | | | | | | | | | |
| 28 |  |  | 26809 | MG028143 | NO | NO | MG366331 |  | | | | | | | | | | | | |
| 28 |  |  | 26810 | MG028144 | MG028413 | NO | MG366332 |  | | | | | | | | | | | | |
| 28 |  |  | 26811 | MG028145 | MG028414 | NO | NO |  | | | | | | | | | | | | |
| 28 |  |  | 26812 | MG028146 | MG028415 | MG100721 | MG366333 |  | | | | | | | | | | | | |
| 29 | Presa Melchor Ocampo | Zacapu | 10431 | MG028147 | MG028416 | MG100722 | MG366334 |  |  | |  | | |  |  | |  | | | |
| 29 |  |  | 10431B | --- | --- | MG100723 | NO |  |  | |  | | |  |  | |  | | | |
| 29 |  |  | 10437 | MG028148 | MG028417 | NO | NO |  | | | | | | | | | | | | |
| 29 |  |  | 10439 | MG028149 | MG028418 | MG100724 | MG366335 |  | | | | | | | | | | | | |
| 29 |  |  | 10440 | MG028150 | MG028419 | NO | MG366336 |  | | | | | | | | | | | | |
| 29 |  |  | 10441 | MG028151 | MG028420 | NO | MG366337 |  | | | | | | | | | | | | |
| 29 |  |  | 10443 | MG028152 | NO | NO | MG366338 |  | | | | | | | | | | | | |
| 29 |  |  | 10444 | MG028153 | MG028421 | NO | MG366339 |  | | | | | | | | | | | | |
| 29 |  |  | 10445 | NO | MG028422 | NO | NO |  | | | | | | | | | | | | |
| 30 | La Zarcita | Zacapu | 25106 | MG028155 | MG028424 | MG100725 | MG366342 |  | | | | | | | | | | | | |
| 30 |  |  | 25106B | --- | --- | NO | MG366343 |  | | | | | | | | | | | | |
| 30 |  |  | 25107 | MG028156 | MG028425 | MG100726 | NO |  | | | | | | | | | | | | |
| 30 |  |  | 25109 | MG028157 | MG028426 | MG100727 | MG366344 |  | | | | | | | | | | | | |
| 30 |  |  | 25110 | MG028158 | MG028427 | MG100728 | MG366345 |  | | | | | | | | | | | | |
| 30 |  |  | 25111 | NO | MG028428 | NO | MG366346 |  | | | | | | | | | | | | |
| 30 |  |  | 25112 | MG028159 | MG028429 | NO | MG366347 |  | | | | | | | | | | | | |
| 30 |  |  | 25113 | MG028160 | MG028430 | NO | MG366348 |  | | | | | | | | | | | | |
| 30 |  |  | 25114 | MG028161 | MG028431 | NO | MG366349 |  | | | | | | | | | | | | |
| 30 |  |  | 25115 | MG028162 | MG028432 | NO | MG366350 |  | | | | | | | | | | | | |
| 30 |  |  | 25116 | MG028163 | MG028433 | NO | MG366351 |  | | | | | | | | | | | | |
| 31 | Laguna de Zacapu | Zacapu | 25102 | MG028154 | MG028423 | NO | MG366340 |  | | | | | | | | | | | | |
| 31 |  |  | 25102B | --- | --- | NO | MG366341 |  | | | | | | | | | | | | |
| 31 |  |  | 26801 | MG028164 | MG028434 | MG100729 | MG366352 |  | | | | | | | | | | | | |
| 31 |  |  | 26802 | MG028165 | MG028435 | NO | MG366353 |  | | | | | | | | | | | | |
| 31 |  |  | 26803 | MG028166 | MG028436 | NO | MG366354 |  | | | | | | | | | | | | |
| 31 |  |  | 26803B | --- | --- | NO | MG366355 |  | | | | | | | | | | | | |
| 31 |  |  | 26804 | MG028167 | MG028437 | NO | MG366356 |  | | | | | | | | | | | | |
| 31 |  |  | 26804B | --- | --- | NO | MG366357 |  | | | | | | | | | | | | |
| 32 | Atenquique | Tamazula | 12012 | MG028188 | MG028455 | MG100748 | NO |  | | | | | | | | | | | | |
| 33 | Puente en Jacona | Bajo Lerma | 17964 | MG028266 | MG028531 | MG100815 | NO |  |  | |  | | |  |  | |  | |  | |
| 33 |  |  | 17965 | MG028267 | MG028532 | MG100816 | MG366468 |  |  | |  | | |  |  | |  | |  | |
| 33 |  |  | 17975 | MG028268 | MG028533 | NO | MG366469 |  |  | |  | | |  |  | |  | |  | |
| 33 |  |  | 17975B | --- | --- | NO | MG366470 |  |  | |  | | |  |  | |  | |  | |
| 33 |  |  | 17976 | MG028269 | MG028534 | MG100817 | MG366471 |  |  | |  | | |  |  | |  | |  | |
| 33 |  |  | 17976B | --- | --- | NO | MG366472 |  |  | |  | | |  |  | |  | |  | |
| 33 |  |  | 17977 | MG028270 | MG028535 | MG100818 | MG366473 |  |  | |  | | |  |  | |  | |  | |
| 34 | Presa La Luz | Bajo Lerma | 39556 | MG028273 | MG028536 | MG100819 | MG366475 |  | | | | | | | | | | | | |
| 34 |  |  | 39557 | MG028271 | MG028537 | MG100820 | MG366476 |  | | | | | | | | | | | | |
| 34 |  |  | 39558 | MG028272 | MG028538 | MG100821 | MG366477 |  | | | | | | | | | | | | |
| 34 |  |  | 39559 | NO | MG028539 | NO | MG366478 |  | | | | | | | | | | | | |
| 34 |  |  | 39560 | MG028276 | MG028540 | NO | NO |  | | | | | | | | | | | | |
| 34 |  |  | 39561 | MG028275 | MG028541 | NO | NO |  | | | | | | | | | | | | |
| 34 |  |  | 39562 | MG028274 | MG028542 | NO | MG366479 |  | | | | | | | | | | | | |
| 34 |  |  | 39563 | MG028277 | MG028543 | MG100822 | MG366480 |  | | | | | | | | | | | | |
| 34 |  |  | 39563B | --- | --- | NO | MG366481 |  | | | | | | | | | | | | |
| 34 |  |  | 39564 | MG028278 | NO | MG100823 | MG366482 |  | | | | | | | | | | | | |
| 34 |  |  | 39564B | --- | --- | MG100824 | NO |  | | | | | | | | | | | | |
| 35 | Quitupan | Balsas | 18739 | MG028208 | MG028473 | MG100764 | MG366410 |  | | | | | | | | | | | | |
| 35 |  |  | 18740 | MG028209 | MG028474 | NO | MG366411 |  | | | | | | | | | | | | |
| 35 |  |  | 18740B | --- | --- | NO | MG366412 |  | | | | | | | | | | | | |
| 35 |  |  | 18745 | MG028210 | MG028475 | NO | MG366413 |  | | | | | | | | | | | | |
| 35 |  |  | 18746 | MG028211 | MG028476 | MG100765 | MG366414 |  | | | | | | | | | | | | |
| 35 |  |  | 18747 | MG028212 | MG028477 | MG100766 | MG366415 |  | | | | | | | | | | | | |
| 35 |  |  | 18747B | --- | --- | MG100767 | NO |  | | | | | | | | | | | | |
| 36 | Presa San Juanico | Cotija | 28133 | MG028213 | MG028478 | NO | MG366416 |  | | | | | | | | | | | | |
| 36 |  |  | 28134 | MG028214 | MG028479 | MG100768 | MG366417 |  | | | | | | | | | | | | |
| 36 |  |  | 28134B | --- | --- | MG100769 | NO |  | | | | | | | | | | | | |
| 36 |  |  | 28135 | MG028215 | MG028480 | MG100770 | MG366418 |  | | | | | | | | | | | | |
| 36 |  |  | 28136 | MG028216 | NO | NO | MG366419 |  | | | | | | | | | | | | |
| 36 |  |  | 28136B | --- | --- | NO | MG366420 |  | | | | | | | | | | | | |
| 36 |  |  | 28137 | MG028217 | NO | MG100771 | MG366421 |  | | | | | | | | | | | | |
| 36 |  |  | 28137B | --- | --- | NO | MG366422 |  | | | | | | | | | | | | |
| 36 |  |  | 28138 | MG028218 | MG028481 | MG100772 | MG366423 |  | | | | | | | | | | | | |
| 36 |  |  | 28139 | MG028219 | MG028482 | MG100773 | MG366424 |  | | | | | | | | | | | | |
| 36 |  |  | 28139B | --- | --- | MG100774 | NO |  | | | | | | | | | | | | |
| 36 |  |  | 28140 | MG028220 | MG028483 | MG100775 | MG366425 |  | | | | | | | | | | | | |
| 36 |  |  | 28164 | MG028221 | MG028484 | MG100776 | MG366426 |  | | | | | | | | | | | | |
| 36 |  |  | 28164B | --- | --- | MG100777 | NO |  | | | | | | | | | | | | |
| 36 |  |  | 28165 | MG028222 | MG028485 | MG100778 | MG366427 |  | | | | | | | | | | | | |
| 36 |  |  | 28166 | MG028223 | MG028486 | MG100779 | MG366428 |  | | | | | | | | | | | | |
| 37 | San Sebastián | Etzatlan-San Marcos | 11920 | MG028168 | MG028438 | MG100730 | MG366358 |  | | | | | | | | | | | | |
| 37 |  |  | 11920B | --- | --- | MG100731 | MG366359 |  | | | | | | | | | | | | |
| 37 |  |  | 11921 | MG028169 | MG028439 | NO | MG366360 |  | | | | | | | | | | | | |
| 37 |  |  | 11921B | --- | --- | NO | MG366361 |  | | | | | | | | | | | | |
| 38 | Presa Palo Verde | Etzatlan-San Marcos | 32485 | MG028175 | MG028443 | MG100735 | MG366371 |  | | | | | | | | | | | | |
| 38 |  |  | 32485B | --- | --- | NO | MG366372 |  | | | | | | | | | | | | |
| 38 |  |  | 32486 | MG028176 | MG028444 | MG100736 | MG366373 |  | | | | | | | | | | | | |
| 38 |  |  | 32487 | MG028177 | MG028445 | MG100737 | NO |  | | | | | | | | | | | | |
| 38 |  |  | 32488 | MG028178 | MG028446 | MG100738 | MG366374 |  | | | | | | | | | | | | |
| 38 |  |  | 32490 | MG028179 | MG028447 | MG100739 | MG366375 |  | | | | | | | | | | | | |
| 38 |  |  | 32491 | MG028180 | MG028448 | MG100740 | MG366376 |  | | | | | | | | | | | | |
| 38 |  |  | 32492 | MG028181 | MG028449 | MG100741 | MG366377 |  | | | | | | | | | | | | |
| 38 |  |  | 32493 | MG028182 | MG028450 | MG100742 | MG366378 |  | | | | | | | | | | | | |
| 38 |  |  | 32494 | MG028183 | NO | NO | NO |  | | | | | | | | | | | | |
| 38 |  |  | 32495 | MG028184 | MG028451 | MG100743 | MG366379 |  | | | | | | | | | | | | |
| 38 |  |  | 32495B | --- | --- | MG100744 | NO |  | | | | | | | | | | | | |
| 38 |  |  | 32577 | MG028185 | MG028452 | MG100745 | MG366380 |  | | | | | | | | | | | | |
| 38 |  |  | 32579 | MG028186 | MG028453 | MG100746 | NO |  | | | | | | | | | | | | |
| 38 |  |  | 32580 | MG028187 | MG028454 | MG100747 | MG366381 |  | | | | | | | | | | | | |
| 39 | San Juanito de Escobedo | Etzatlan-San Marcos | 28171 | MG028170 | MG028440 | NO | MG366362 |  | | | | | | | | | | | | |
| 39 |  |  | 28171B | --- | --- | NO | MG366363 |  | | | | | | | | | | | | |
| 39 |  |  | 28172 | MG028171 | NO | MG100732 | MG366364 |  | | | | | | | | | | | | |
| 39 |  |  | 28173 | MG028172 | MG028441 | MG100733 | MG366365 |  | | | | | | | | | | | | |
| 39 |  |  | 28173B | --- | --- | NO | MG366366 |  | | | | | | | | | | | | |
| 39 |  |  | 28174 | MG028173 | NO | NO | MG366367 |  | | | | | | | | | | | | |
| 39 |  |  | 28174B | --- | --- | NO | MG366368 |  | | | | | | | | | | | | |
| 39 |  |  | 28175 | MG028174 | MG028442 | MG100734 | MG366369 |  | | | | | | | | | | | | |
| 39 |  |  | 28175B | --- | --- | NO | MG366370 |  | | | | | | | | | | | | |
| 40 | Cuescomatitlán | Grande de Santiago | 5094 | MG028244 | MG028509 | MG100798 | MG366441 |  | | | | | | | | | | | | |
| 40 |  |  | 5094B | --- | --- | MG100799 | MG366442 |  | | | | | | | | | | | | |
| 40 |  |  | 5095 | MG028245 | MG028510 | NO | MG366443 |  | | | | | | | | | | | | |
| 40 |  |  | 5096 | MG028246 | MG028511 | NO | MG366444 |  | | | | | | | | | | | | |
| 40 |  |  | 5097 | MG028247 | MG028512 | MG100800 | MG366445 |  | | | | | | | | | | | | |
| 40 |  |  | 5097B | --- | --- | NO | MG366446 |  | | | | | | | | | | | | |
| 40 |  |  | 5098 | MG028248 | MG028513 | NO | MG366447 |  | | | | | | | | | | | | |
| 40 |  |  | 5098B | --- | --- | NO | MG366448 |  | | | | | | | | | | | | |
| 41 | Jalpa | Grande de Santiago | 5769 | MG028249 | MG028514 | NO | MG366449 |  | | | | | | | | | | | | |
| 41 |  |  | 20875 | MG028250 | MG028372 | MG100801 | MG366450 |  | | | | | | | | | | | | |
| 41 |  |  | 20875B | --- | --- | MG100802 | NO |  | | | | | | | | | | | | |
| 42 | San Antonio | Grande de Santiago | 36290 | MG028251 | MG028515 | MG100803 | MG366451 |  | | | | | | | | | | | | |
| 42 |  |  | 36290B | --- | --- | MG100804 | MG366452 |  | | | | | | | | | | | | |
| 42 |  |  | 36291 | MG028252 | MG028516 | MG100805 | MG366453 |  | | | | | | | | | | | | |
| 42 |  |  | 36291B | --- | --- | MG100806 | MG366454 |  | | | | | | | | | | | | |
| 43 | Presa de Garabato | Grande de Santiago | 36314 | MG028253 | MG028517 | MG100807 | MG366455 |  | | | | | | | | | | | | |
| 43 |  |  | 36314B | --- | --- | NO | MG366456 |  | | | | | | | | | | | | |
| 43 |  |  | 36315 | MG028254 | MG028518 | NO | MG366457 |  | | | | | | | | | | | | |
| 43 |  |  | 36315B | --- | --- | NO | MG366458 |  | | | | | | | | | | | | |
| 43 |  |  | 36319 | MG028255 | MG028519 | MG100808 | MG366459 |  | | | | | | | | | | | | |
| 43 |  |  | 36319B | --- | --- | NO | MG366460 |  | | | | | | | | | | | | |
| 43 |  |  | 36320 | MG028256 | MG028521 | MG100809 | MG366461 |  | | | | | | | | | | | | |
| 43 |  |  | 36320B | --- | --- | MG100810 | NO |  | | | | | | | | | | | | |
| 43 |  |  | 36322 | MG028257 | MG028520 | MG100811 | MG366462 |  | | | | | | | | | | | | |
| 43 |  |  | 36322B | --- | --- | NO | MG366463 |  | | | | | | | | | | | | |
| 43 |  |  | 36323 | MG028258 | MG028522 | MG100812 | MG366464 |  | | | | | | | | | | | | |
| 43 |  |  | 36323B | --- | --- | NO | MG366465 |  | | | | | | | | | | | | |
| 44 | Río Tinajeros | Grande de Santiago | 36324 | MG028259 | MG028523 | MG100813 | MG366466 |  | | | | | | | | | | | | |
| 44 |  |  | 36324B | --- | --- | MG100814 | MG366467 |  | | | | | | | | | | | | |
| 44 |  |  | 36326 | MG028260 | MG028524 | NO | NO |  | | | | | | | | | | | | |
| 44 |  |  | 36327 | MG028261 | MG028525 | NO | NO |  | | | | | | | | | | | | |
| 44 |  |  | 36328 | MG028262 | MG028526 | NO | NO |  | | | | | | | | | | | | |
| 44 |  |  | 36329 | MG028263 | MG028527 | NO | NO |  | | | | | | | | | | | | |
| 44 |  |  | 36330 | MG028264 | MG028528 | NO | NO |  | | | | | | | | | | | | |
| 44 |  |  | 36331 | MG028265 | MG028529 | NO | NO |  | | | | | | | | | | | | |
| 44 |  |  | 36332 | NO | MG028530 | NO | NO |  | | | | | | | | | | | | |
|  | *Poeciliopsis prolifica* |  | 42668 | MG028009 | MG028279 | MG100617 | MG366191 |  | | | | | | | | | | | | |
|  | *Poeciliopsis prolifica* |  | 42668B | --- | --- | NO | MG366192 |  | | | | | | | | | | | | |
|  | *Poeciliopsis prolifica* |  | 42669 | MG028010 | MG028280 | MG100618 | MG366193 |  | | | | | | | | | | | | |
|  | *Poeciliopsis prolifica* |  | 42670 | MG028011 | MG028281 | MG100619 | NO |  | | | | | | | | | | | | |
|  | *Poeciliopsis prolifica* |  | 42670B | --- | --- | MG100620 | NO |  | | | | | | | | | | | | |
|  | *Poeciliopsis prolifica* |  | 42671 | MG028012 | MG028282 | MG100621 | NO |  | | | | | | | | | | | | |
|  | *Poeciliopsis prolifica* |  | 42812 | MG028013 | MG028283 | NO | NO |  | | | | | | | | | | | | |
|  | *Poeciliopsis prolifica* |  | 42815 | MG028014 | MG028284 | NO | NO |  | | | | | | | | | | | | |
|  | *Poeciliopsis prolifica* |  | 42816 | MG028015 | MG028285 | NO | NO |  | | | | | | | | | | | | |
|  | *Poeciliopsis prolifica* |  | 42817 | MG028016 | MG028286 | NO | NO |  | | | |  | | | | | | | | |
|  | *Poeciliopsis prolifica* |  | 42818 | MG028017 | MG028287 | NO | NO |  | | | | | | | | | | | | |
|  | *Poeciliopsis prolifica* |  | 42820 | MG028018 | MG028289 | NO | NO |  | | | | | | | | | | | | |
|  | *Poeciliopsis prolifica* |  | 42821 | MG028019 | MG028290 | NO | NO |  | | | | | | | | | | | | |
|  | *Poeciliopsis prolifica* |  | 42822 | MG028020 | MG028291 | NO | MG366194 |  | | | | | | | | | | | | |
|  | *Poeciliopsis prolifica* |  | 42822B | --- | --- | NO | MG366195 |  | | | | | | | | | | | | |
|  | *Poeciliopsis prolifica* |  | 42824 | MG028021 | MG028292 | NO | NO |  | | | | | | | | | | | | |
|  | *Poeciliopsis prolifica* |  | 42825 | MG028022 | MG028293 | NO | NO |  | | | | | | | | | | | | |
