# Supplementary material for: Evolving in the highlands: the case of the Neotropical Lerma live-bearing Poeciliopsis infans (Woolman, 1894) (Cyprinodontiformes: Poeciliidae) in Central Mexico
Source: BMC Evol Biol. 2018 Apr 20;18:56. doi: 10.1186/s12862-018-1172-7 (PMC5910627; doi:10.1186/s12862-018-1172-7)
Supplement: Supplementary file 2 — Primers, PCR conditions, and References. (DOC 35 kb) [file 12862_2018_1172_MOESM2_ESM.doc]

Additional file 2. Primers, PCR conditions, and References.

|  | *Cytb* | *coxI* | *S7* | *RHO* |
| --- | --- | --- | --- | --- |
| Primers | Glu-F  Thr-R | Fish-F1  Fish-R1 | S7RPEX1F  S7RPEX3R | RH193F  RH1073R |
| Size (pb) | 533 | 626 | 859 | 845 |
| Reference | Doadrio and  Domínguez, (2004). | Ward *et al*.  (2005). | Chow & Hazama, (1998) | Chen, Bonillo & Lecointre (2003) |
| Denaturing  (step 1) | 94ºC, 2 min. | 94ºC, 2 min. | 94ºC, 3 min. | 94ºC, 3 min. |
| Cycles  (step 2) | 35 | 35 | 32 | 32 |
| Denaturing | 94ºC, 45 s. | 94ºC, 30 s. | 94ºC, 45 s. | 94ºC, 45 s. |
| Annealing | 47ºC, 1 min. | 50ºC, 30 s. | 58.5ºC, 30 s. | 55ºC, 45 s. |
| Extension | 72ºC, 90 s. | 72ºC, 1 min. | 72ºC, 90 seg. | 72ºC, 90 s. |
| Final Extension (step 3) | 72ºC, 5 min. | 72ºC, 10 min. | 72ºC, 7 min. | 72ºC, 7 min. |
