# Supplementary material for: Evolving in the highlands: the case of the Neotropical Lerma live-bearing Poeciliopsis infans (Woolman, 1894) (Cyprinodontiformes: Poeciliidae) in Central Mexico
Source: BMC Evol Biol. 2018 Apr 20;18:56. doi: 10.1186/s12862-018-1172-7 (PMC5910627; doi:10.1186/s12862-018-1172-7)
Supplement: Supplementary file 3 — Models selected with Akaike information criterion and the parameters of each gene. (DOC 32 kb) [file 12862_2018_1172_MOESM3_ESM.doc]

Additional file 3. Models selected with Akaike information criterion and the parameters of each gene.

|  | *Cytb* | *coxI* | *S7* | *RHO* |
| --- | --- | --- | --- | --- |
| Model | GTR+I+G | GTR+I | TVM+I+G | TrN+I+G |
| Frec. A | 0.2457 | 0.2488 | 0.2802 | 0.1890 |
| Frec. C | 0.3046 | 0.2696 | 0.1863 | 0.2900 |
| Frec. G | 0.1469 | 0.1718 | 0.2347 | 0.2276 |
| Frec. T | 0.3029 | 0.3098 | 0.2988 | 0.2935 |
| P-inv | 0.0000 | 0.8300 | 0.1600 | 0.0000 |
| Gamma shape | 0.1220 | N/A | 0.0250 | 0.9983 |
