# Supplementary material for: Evolving in the highlands: the case of the Neotropical Lerma live-bearing Poeciliopsis infans (Woolman, 1894) (Cyprinodontiformes: Poeciliidae) in Central Mexico
Source: BMC Evol Biol. 2018 Apr 20;18:56. doi: 10.1186/s12862-018-1172-7 (PMC5910627; doi:10.1186/s12862-018-1172-7)
Supplement: Supplementary file 4 — Geographical coordinates of the 162 sites registers in Colección de Peces de la Universidad Michoacana de San Nicolás de Hidalgo used as presence data for species distribution modelling. (DOC 161 kb) [file 12862_2018_1172_MOESM4_ESM.doc]

Additional file 4. Geographical coordinates of the 162 sites registers in Colección de Peces de la Universidad Michoacana de San Nicolás de Hidalgo used as presence data for species distribution modelling.

| **Locality** | **Latitude** | **Longitude** |
| --- | --- | --- |
| 4 mi S San Miguel de Allende | 20.850226 | -100.793422 |
| below laguna de Naranja | 19.7813659 | -101.762748 |
| 1 km downstream Platanal | 19.9346 | -102.252 |
| 1 mi ESE of Teuchitlan | 20.67878 | -103.838685 |
| 1 mi WNW of Cuyutlan | 20.424082 | -103.375023 |
| 2 km S of Magdalena | 20.89278 | -104.031065 |
| 2.5 mi S of San Marcos | 20.3 | -103.533333 |
| 200 m above main bridge into Compostela | 21.231934 | -104.900894 |
| 25 m offshore of Manantial Ojo de la Liebre | 19.8222613 | -101.788056 |
| 25 mi E of Ameca | 20.687398 | -103.693749 |
| 3.7 mi E Atequiza | 20.403876 | -103.070958 |
| 5 km W of Teuchitlan | 20.69267 | -103.91669 |
| 5 mi N of Yahualica | 21.2299 | -102.851 |
| 5 mi NE Piedad | 20.407505 | -101.956027 |
| 5 mi W of Ameca | 20.481476 | -103.975073 |
| 6 de Enero | 21.52464 | -104.80384 |
| 6 mi N Colimilla | 20.691996 | -103.228296 |
| 7 mi E of Penjamo | 20.4532116 | -101.607094 |
| Above & below the presa at Copalita | 20.838393 | -103.419 |
| Achacales | 19.70577 | -104.14599 |
| Andocutin | 19.9384243 | -100.854608 |
| Angamacutiro | 20.1446232 | -101.704673 |
| Apaseo El Grande | 20.5396064 | -100.696937 |
| Araro,manantiales al E Cuitzeo | 19.9071176 | -100.831779 |
| Arroyo en carr. A la Estancia Km 1 | 21.4100833 | -102.7375 |
| Arroyo en Quitupan | 19.9243333 | -102.871972 |
| Arroyo en San Carlos | 20.7852222 | -102.766306 |
| Arroyo San Andres | 20.780819 | -104.181026 |
| Arroyo San Gabriel | 21.128684 | -100.861203 |
| Atotonilco | 21.0021667 | -100.799472 |
| Balneario at Tocumbo | 19.703312 | -102.514432 |
| Balneario Atotonilco | 20.5613871 | -102.510781 |
| Balneario Chorros de Tala | 20.69547 | -103.67935 |
| Balneario El Rincon | 20.689558 | -103.841509 |
| below dam for Presa Ignacio Allende | 20.840874 | -100.828553 |
| between Etzatlan and San Marcos | 20.7776 | -104.164 |
| between Ocotlan and Chapala | 20.387814 | -102.824525 |
| Bordo cerca de Chimaliquin | 21.3566667 | -102.801889 |
| bridge at Estanzuela | 20.5243611 | -104.336667 |
| bridge between Zamora and Jacona | 19.972381 | -102.295393 |
| Canal at Tarecuato | 19.8436 | -102.479261 |
| Canal de Querendaro 2 | 19.8682102 | -100.972431 |
| Canal en Rancho Nuevo | 20.7008056 | -102.939583 |
| Capacho | 19.9613278 | -101.224772 |
| Carretera 304 San Julian-San Miguel el Alto | 21.0088056 | -102.296639 |
| Chiquimitio | 19.799 | -101.246083 |
| Cienega en La Purisima | 19.5221667 | -103.342472 |
| Cieneguilla | 20.9522222 | -100.795194 |
| Copandaro | 19.899473 | -101.216961 |
| Copandaro de Jimenez | 19.8898489 | -101.668758 |
| Desembocadura del Rio Querendaro | 19.9009528 | -100.976 |
| ditch 2 mi N Guadalajara | 20.75 | -103.366667 |
| ditch 2.5 N Etzatlan | 20.797845 | -104.094427 |
| ditch 8 mi by hwy 70 east of Ameca | 20.546389 | -103.907617 |
| ditch between Ameca & Hwy 15 | 20.591616 | -103.828087 |
| Dren la Cinta | 20.0860194 | -101.154617 |
| E edge of Belen del Refugio | 21.530129 | -102.432634 |
| El Borbollon, La Maiza | 19.5026833 | -101.384592 |
| El Chacalito | 19.8129333 | -104.240933 |
| El Nacimiento | 20.5403286 | -100.613519 |
| El Palo Verde reservoir | 20.769055 | -104.11573 |
| El Parian | 19.6892194 | -101.26838 |
| Estacion Querendaro | 19.8823167 | -100.931714 |
| exhacienda de Guadalupe | 19.6589726 | -101.273074 |
| Ferrocarrileros | 20.8126389 | -100.818611 |
| Granja Sanhuarripa | 20.7770249 | -104.163603 |
| Hacienda San Sebastian | 20.822912 | -104.119572 |
| Hwy 54 bridge in Apozol | 21.476142 | -103.088792 |
| Iramuco | 19.9577778 | -100.923056 |
| Irrigation canal 3.7 mi NE Alvaro Obregon | 19.806554 | -101.063069 |
| La Canal, on E of Tocumbo | 19.7033 | -102.519 |
| La Mintzita | 19.644973 | -101.274336 |
| La Vega | 20.5833333 | -103.85 |
| Lago de Atotonilco | 20.3603 | -103.654 |
| Lago de Camecuaro | 19.902411 | -102.209455 |
| Lago de Chapala | 20.279166 | -103.1875 |
| Lago de Cuitzeo | 19.9628975 | -101.059816 |
| Lago de Magdalena | 20.902806 | -104.017245 |
| Lago de Zapotlan | 19.748632 | -103.469951 |
| Laguna Colorada | 20.756772 | -103.989213 |
| Laguna Corralejo | 20.5127318 | -101.607914 |
| Laguna de Cajititlan | 20.415926 | -103.329852 |
| Laguna de Sayula | 20.075605 | -103.509025 |
| Laguna de Yuriria | 20.241507 | -101.203946 |
| Laguna de Zacapu | 19.823495 | -101.787291 |
| Laguna San Marcos | 20.29057 | -103.551612 |
| Laguna Zacoalco | 20.243351 | -103.589273 |
| Las Adjuntas | 20.670525 | -101.859447 |
| Los Cipreses | 19.827634 | -101.787137 |
| Los Lavaderos | 19.8835722 | -100.447078 |
| Los Negritos | 20.061113 | -102.609368 |
| Manantial Bellas Fuentes | 19.8215836 | -101.68013 |
| Manantial en San Jose de Gracia | 20.6742222 | -102.555389 |
| Manantial in Quinceo | 19.7339767 | -101.222332 |
| Manantial La Luz | 19.9370475 | -102.299697 |
| Manantial Orandino | 19.956206 | -102.325994 |
| Manantial San Cristobal | 19.9610861 | -101.315353 |
| Manantial San Francisco del Rincon | 21.0512833 | -101.844344 |
| Molino viejo cerca de la Cofradia | 20.3898611 | -103.755028 |
| N Tanhuato | 20.291134 | -102.329762 |
| near Balneario El Cortijo | 20.992746 | -100.797794 |
| near Cuyacapan | 19.95954 | -103.51704 |
| near Estancia de Ayones | 20.901222 | -104.079639 |
| Ojo de Agua de Santiaguito | 21.046216 | -101.835825 |
| Palo Blanco at bridge crossing | 19.7417 | -104.178 |
| Parque La Angostura | 19.8277229 | -101.787093 |
| Peninsula de San Agustin del Pulque | 19.9533389 | -101.109139 |
| Petatan, Lago de Chapala | 20.1626944 | -102.867944 |
| Pond at end of Lago Union de Tula | 19.940254 | -104.257415 |
| Presa Aristeo Mercado | 19.929525 | -101.669319 |
| Presa Buenavista | 20.3349722 | -103.755611 |
| Presa de Cointzio | 19.616234 | -101.280209 |
| Presa de Garabato | 20.6245556 | -102.687667 |
| Presa de Huapango | 19.9246389 | -99.8017778 |
| Presa El Alamo | 20.1986413 | -99.7867317 |
| Presa el Pajonal | 19.539375 | -101.417772 |
| Presa Ignacio Allende | 20.8507052 | -100.823794 |
| Presa Juriquilla | 20.7039699 | -100.462281 |
| Presa Melchor Ocampo | 20.0945908 | -101.738532 |
| Presa Palote | 21.18 | -101.68944 |
| Presa San Antonio de Huaracha | 19.962975 | -102.578083 |
| Presa San Ignacio | 20.6357222 | -103.931733 |
| Presa San Juanico | 19.847777 | -102.685833 |
| Presa Teclan | 20.3205 | -103.734861 |
| Presa Valle de Juarez | 19.9385556 | -102.949719 |
| Pueblo Rio Laja | 21.20625 | -100.922333 |
| Puente Chapulimita | 20.68025 | -103.908139 |
| Ribera del Lago de Cuitzeo, 2.5 km al norte del Salitre | 19.9167444 | -101.299483 |
| Rinconcillo | 20.7889444 | -100.806944 |
| Rio Ameca at Ameca | 20.543587 | -104.043992 |
| Rio Ameca en San Blasito | 20.7038139 | -104.309581 |
| Rio Calvillo | 21.847038 | -102.739145 |
| Rio Celio | 19.947655 | -102.304475 |
| Rio chiquito de Amatlan de Cañas | 20.8026417 | -104.417678 |
| Rio de la Laja below bridge | 20.670345 | -100.751515 |
| Rio Duero, 9.2 mi W Chilchota | 19.912489 | -102.205813 |
| Rio Grande, north of Zacapu | 19.824226 | -101.77389 |
| Rio juchipila en el poblado de jalpa | 21.6518 | -102.966 |
| Rio Juchipila N Santa Rosa | 21.602895 | -102.94872 |
| Rio la Patera | 19.9211278 | -101.724569 |
| Rio Salado | 20.68675 | -103.693361 |
| Rio Santiago at Poncitlan | 20.38502 | -102.923955 |
| Rio Teuchitlan, E edge of Teuchitlan | 20.682196 | -103.843366 |
| Rio Tizapan | 20.0431667 | -103.803722 |
| Rio Turbio | 20.75 | -101.833333 |
| Rio Turbio, 8 mi E Penjamo | 20.4532 | -101.607 |
| Rio Tuxpan, 1 km N of Atenquique | 19.530125 | -103.430328 |
| Rio Xoconoxtle San Juan | 20.9420833 | -100.977222 |
| Rio Zula bajo el puente | 20.4131911 | -102.724989 |
| Road by La Quemada | 21.3271944 | -101.095806 |
| S Empalme Escobedo | 20.597196 | -100.746606 |
| San Nicolas, Rio Verde | 21.29595 | -102.549917 |
| SE corner, in Yahualica | 21.167672 | -102.881113 |
| Spring-fed pond N Jaripo | 19.942262 | -102.591945 |
| Stream E El Refugio de los Orendain | 20.725792 | -103.64856 |
| stream flowing into a presa near La Quemada | 20.9631056 | -104.052878 |
| Stream near Santa Anna | 20.539257 | -103.434305 |
| Stream near Santa Cruz de las Flores | 20.469271 | -103.506936 |
| Tarejero Spring | 19.8215407 | -101.717526 |
| Texcalme | 20.4580944 | -104.070367 |
| Tierra colorada | 19.7998806 | -101.247481 |
| Trib to Ameca W of Ameca | 20.535054 | -104.057926 |
