# Supplementary material for: Evolving in the highlands: the case of the Neotropical Lerma live-bearing Poeciliopsis infans (Woolman, 1894) (Cyprinodontiformes: Poeciliidae) in Central Mexico
Source: BMC Evol Biol. 2018 Apr 20;18:56. doi: 10.1186/s12862-018-1172-7 (PMC5910627; doi:10.1186/s12862-018-1172-7)
Supplement: Supplementary file 5 — Ambiguously Aligned Regions for S7. (DOC 49 kb) [file 12862_2018_1172_MOESM5_ESM.doc]

Additional file 5. Ambiguously Aligned Regions for *S7.*

|  | **BP** | | | | | | | | | | | | | | | | | | | |
| --- | --- | --- | --- | --- | --- | --- | --- | --- | --- | --- | --- | --- | --- | --- | --- | --- | --- | --- | --- | --- |
| **Clade** | 244 | 392 | 402 | 403 | 404 | 410 | 411 | 417 | 418 | 419 | 420 | 431 | 456 | 457 | 458 | 471 | 472 | 504 | 505 | 511 |
| A1 | C | -/T | A | A | -/A | -/T | A | T | -/T | A | A | T | A | T | G | G | C | A | G | C |
| A2 | C | -/T | A | A | -/A | -/T | -/A | T | -/T/G | A | A | -//T | -/A | -/T | -/G | -/G | -/C | -/A | -/G | -/C |
| A3 | C | -/T | -/A | -/A | -/A | -/T | A | -/T | -/T/G | -/A | -/A | T | A | T | G | G | C | A | G | C |
| B | - | -/T | - | - | - | - | A | -/T | -/G | -/A | -/A | T | A | T | G | G | C | A | G | C |

|  | **BP** | | | | | | | | | | | | | | | | | | | |
| --- | --- | --- | --- | --- | --- | --- | --- | --- | --- | --- | --- | --- | --- | --- | --- | --- | --- | --- | --- | --- |
| **Clade** | 512 | 605 | 606 | 607 | 608 | 609 | 610 | 613 | 614 | 615 | 616 | 617 | 618 | 619 | 620 | 621 | 622 | 623 | 624 | 625 |
| A1 | A | -/A | -/T | -/T | -/A | -/G | -/C | T | A | G | C | T | T | A | A | G | C | T | A | G |
| A2 | -/A/T | A | T | T | A | G | C | -/T | -/A | -/G | -/C | -/T | -/T | A | A | G | C | T | A | G |
| A3 | T/A | A | T | T | A | G | C | -/T | A | -/G | -/C | -/T | -/T | -/A | -/A | -/G | -/C | -/T | -/A | -/G |
| B | A | A | T | T | A | G | C | T | A | G | C | T | T | A | A | G | C | T | A | G |

|  | **BP** | | | | | | | | | | | | |
| --- | --- | --- | --- | --- | --- | --- | --- | --- | --- | --- | --- | --- | --- |
| **Clade** | 626 | 627 | 628 | 629 | 644 | 708 | 709 | 710 | 711 | 781 | 782 | 785 | 786 |
| A1 | A | C | G | A | A | A | C | A | T | C | A | G | C |
| A2 | -/A | -/C | -/G | -/A | -/A | A | C | A | T | C | A | G | C |
| A3 | -/A | -/C | -/G | A | A | A | C | A | T | C | A | G | C |
| B | A | C | G | A | A | -/A | -/C | -/A | -/T | -/C | -/A | -/G | -/C |
