# Supplementary material for: Evolving in the highlands: the case of the Neotropical Lerma live-bearing Poeciliopsis infans (Woolman, 1894) (Cyprinodontiformes: Poeciliidae) in Central Mexico
Source: BMC Evol Biol. 2018 Apr 20;18:56. doi: 10.1186/s12862-018-1172-7 (PMC5910627; doi:10.1186/s12862-018-1172-7)
Supplement: Supplementary file 10 — The Bayesian inference tree of P. infans from S7 nuclear gene (859 bp). Bayesian posterior probability (> 0.9; above the branches) and maximum likelihood bootstrap values (> 80%; below the branches) are indicated. (DOC 715 kb) [file 12862_2018_1172_MOESM10_ESM.doc]

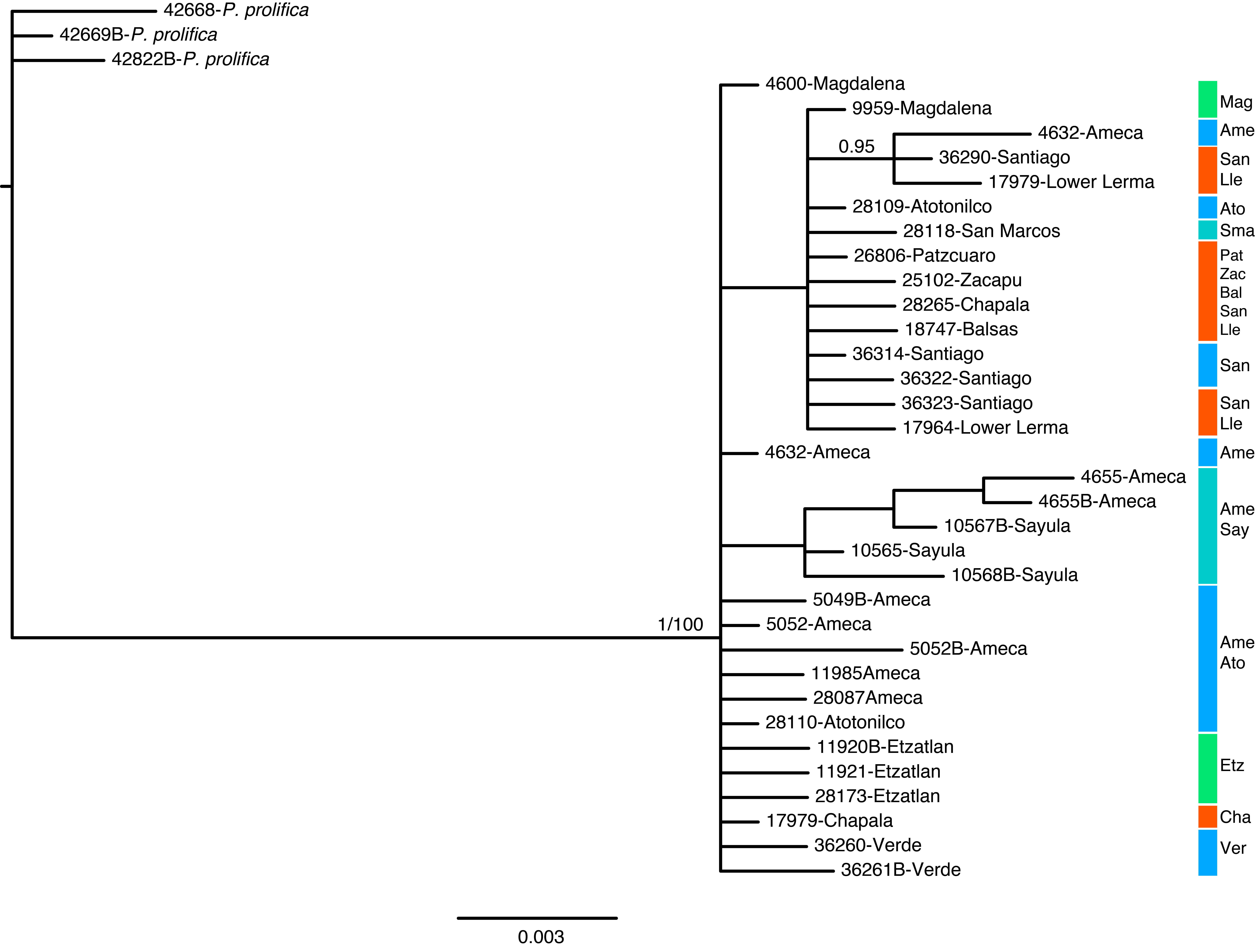


Additional file 10. The Bayesian inference tree of *P. Infans* from *S7* nuclear gene (859 bp). Bayesian posterior probability (>0.9; before the diagonal) and maximum likelihood bootstrap values (>80%; after the diagonal) are indicated.
