# Supplementary material for: Evolving in the highlands: the case of the Neotropical Lerma live-bearing Poeciliopsis infans (Woolman, 1894) (Cyprinodontiformes: Poeciliidae) in Central Mexico
Source: BMC Evol Biol. 2018 Apr 20;18:56. doi: 10.1186/s12862-018-1172-7 (PMC5910627; doi:10.1186/s12862-018-1172-7)
Supplement: Supplementary file 11 — The Bayesian inference tree of P. infans from concatenated sequences of two nuclear genes (S7 and RHO: 1704 bp). Bayesian posterior probability (> 0.9) and maximum likelihood bootstrap values (> 80%) are indicated. (DOC 2382 kb) [file 12862_2018_1172_MOESM11_ESM.doc]

Additional file 11. The Bayesian inference tree of *P. Infans* from concatenated sequences of two nuclear genes (*S7* and *RHO:* 1,704 bp). Bayesian posterior probability (>0.9) and maximum likelihood bootstrap values (>80%) are indicated.
