# Supplementary material for: Evolving in the highlands: the case of the Neotropical Lerma live-bearing Poeciliopsis infans (Woolman, 1894) (Cyprinodontiformes: Poeciliidae) in Central Mexico
Source: BMC Evol Biol. 2018 Apr 20;18:56. doi: 10.1186/s12862-018-1172-7 (PMC5910627; doi:10.1186/s12862-018-1172-7)
Supplement: Supplementary file 12 — Haplotype networks for nuclear genes, a) S7 gene, b) RHO gene. The two recovered haplogroups are show with labels A and B. (DOC 1189 kb) [file 12862_2018_1172_MOESM12_ESM.doc]

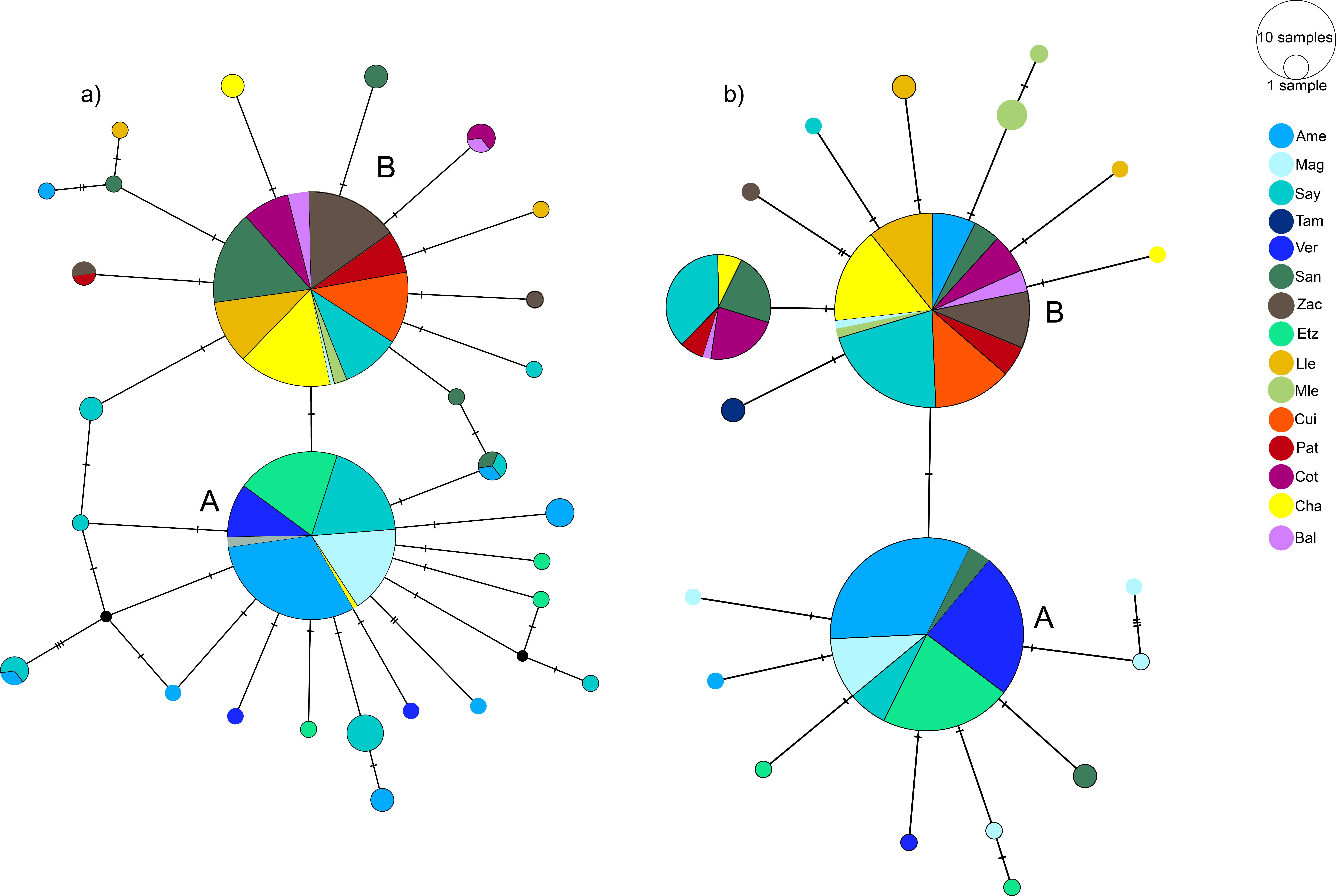


Additional file 12. Haplotype networks for nuclear genes, a) *S7* gene, b) *RHO* gene. The two recovered haplogroups are show with labels A and B.
